# A systematic review of clinicians’ acceptance and use of clinical decision support systems over time

**DOI:** 10.1038/s41746-025-01662-7

**Published:** 2025-05-26

**Authors:** Nicki Newton, Adeola Bamgboje-Ayodele, Rowena Forsyth, Amina Tariq, Melissa T. Baysari

**Affiliations:** 1 Digital Health Human Factors Research Group, Sydney Nursing School, Faculty of Medicine and Health, The University of Sydney, Sydney, NSW Australia; 2 Discipline of Design, School of Architecture, Design and Planning, The University of Sydney, Sydney, Australia; 3 Biomedical Informatics and Digital Health, School of Medical Sciences, Faculty of Medicine and Health, The University of Sydney, Sydney, NSW Australia; 4 Australian Centre for Health Services Innovation and Centre for Healthcare Transformation, School of Public Health and Social Work, Faculty of Health, Queensland University of Technology, Brisbane, QLD Australia

**Keywords:** Human behaviour, Health services, Software, Decision making

## Abstract

Existing reviews have identified factors influencing Clinical Decision Support (CDS) adoption by clinicians in practice but overlook the dynamic and evolving nature of technology and users’ needs over time. This review aimed to identify factors that influence early, mid-term, and sustained acceptance and use of CDS in hospital settings. Five databases were searched from 2007 to January 2024 and 67 papers were included. Factors were extracted and synthesised according to the time that data were collected following CDS implementation. Factors relating to the CDS intervention (e.g. utility) and inner setting (e.g. fit with workflows) were reported across all time periods. Perceived outcomes were more often identified in the first year of use, and individual factors after the first 6 months of use. Strategies to work around CDS limitations were reported 5 years after implementation. Our review provides guidance for developing, implementing, and supporting ongoing use of CDS systems.

## Introduction

Clinical Decision Support (CDS) systems offer many opportunities to improve patient care in hospitals^1^. However, the impact of CDS on workflows and clinical outcomes is generally reported to be low in practice^2–4^. Clinicians’ uptake of CDS, an essential step to realising these outcomes, was recently reported to be just 34.2% in a meta-analysis conducted across 60 CDS study arms^5^.

Existing reviews have identified the factors that influence CDS success in depth, providing insight into why some CDS systems are more likely to be used by clinicians than others^6–10^. Factors commonly reported include the systems’ usefulness and ease of use, its fit with existing workflows, and the provision of resources to support users^7,10^. However, existing evidence syntheses have conceptualised these factors in a static, cross-sectional nature that assumes they remain equally relevant from clinicians’ initial uptake of CDS through to routine, sustained use. This assumption has been challenged in several studies. For example, one study found clinicians’ perceptions corresponded to their level of exposure to CDS^11^ and another found different issues were relevant to clinicians at different points in time following implementation of a system containing decision support features^12^. Taken together with theories of technology adoption, such as the diffusion of innovations theory^13,14^ and normalisation process theory^15^, that describe the temporal nature of embedding complex interventions into routine practice, evidence indicates that CDS use is likely to be a dynamic process where user needs unfold and change over time.

Understanding factors that influence acceptance and use of CDS across the system lifecycle would allow for the deployment of targeted, adaptive, and relevant strategies to anticipate user needs, encouraging both initial uptake and sustained use over time^5^. However, this has not yet been systematically examined. The current study aimed to address this gap by systematically reviewing the literature to identify factors that influence early, mid-term, and sustained acceptance and use of CDS in hospital settings.

## Results

**Fig. 1:**
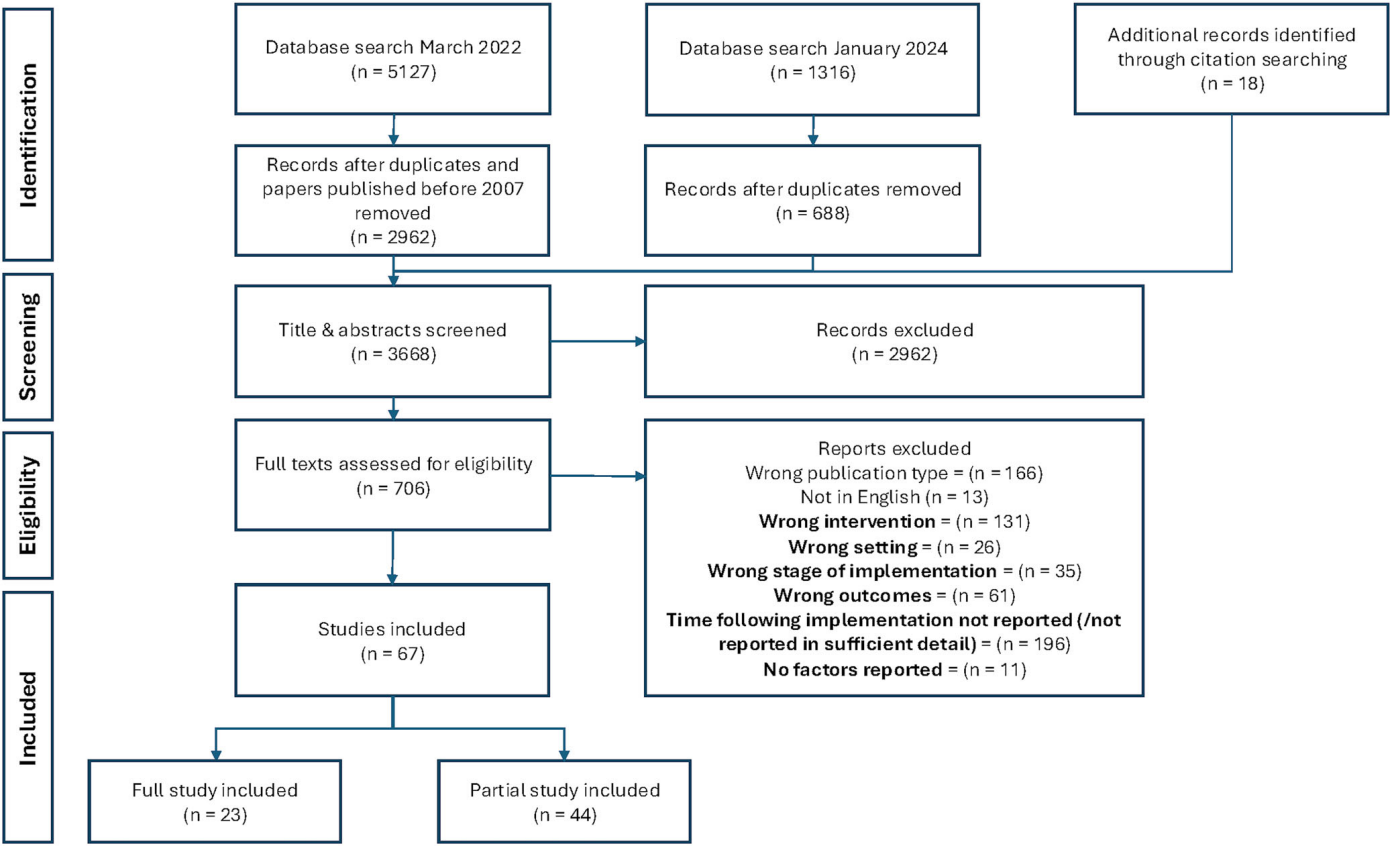
PRISMA flow diagram.

A flowchart of the search strategy, selection process and exclusions is presented in Fig. 1. Out of 67 studies included in the review, 23 studies (34%) contained entirely relevant results and all results were extracted from these studies for analysis. Forty four studies (66%) contained partially relevant results, i.e. some results met inclusion criteria and were extracted for analysis, while other results were excluded from analysis. Common reasons for excluding results are detailed in Supplementary Table 2.

### Study characteristics and methods

Study characteristics and methods are presented in Supplementary Table 4. Over half of the studies were conducted in the United States (*n* = 38), with remaining studies conducted across 17 other countries (Supplementary Table 4). Methods used to evaluate clinicians’ acceptance and use of CDS included surveys and questionnaires (52%), interviews (33%), analysis of system data (19%), focus groups (12%) and observations (7%). Twelve studies used two or more methods at the same point in time, with interviews and observations being the most common pairing (*n* = 5).

### Interventions

CDS interventions were heterogeneous, with interruptive alerts targeting medication management being the most common form of CDS evaluated (*n* = 14) (see Table 1). Forty-five studies evaluated knowledge-based CDS systems (i.e. guideline, rule or algorithm based), 13 studied non-knowledge (i.e. AI or ML based systems), 2 evaluated CDS with knowledge and non-knowledge-based components and 7 studies did not report CDS to this level of detail (Table 1).

**Table 1:** Study interventions

Timeframe	First author	Year	CDS type	Knowledge or non-knowledge based CDS	CDS target	CIS details and level of integration*
0–1 months	Castellanos^16^,^a^	2018	Passive recommendations	Knowledge	Appropriate procalcitonin testing for sepsis	Off the shelf, unidirectional data exchange, embedded in CIS
	Grauer^17^	2022	Interruptive alerts and indications	Knowledge	Indications for medication orders	Off the shelf, bidirectional, embedded
1–2 months	Castellanos^16^^*,*a^	2018	*(as above)*			
	Guidi^18^	2015	Interruptive alerts	Knowledge	Early warning system to detect patients at risk for sudden clinical deterioration and development of severe sepsis	Off the shelf, bidirectional, embedded in CIS
	Sauro^19^^*,*a^	2019	Order set	Knowledge	Increase use of low molecular weight heparin for VTE prophylaxis	Off the shelf, bidirectional data exchange, embedded in CIS
	Tsai^20^	2022	Dashboard	Non-knowledge	Monitoring prognostic risk across 8 different diseases	NR, unidirectional, NR
2–3 months	Castellanos^16^^*,*a^	2018	*(as above)*			
	DeBie^21^	2021	Dynamic checklist	Non-knowledge	Supporting ICU ward rounds	NR, unidirectional data exchange, NR
	Harrison^22^	2017	Passive alerts	Non-knowledge	Detection of severe sepsis	Developed, bidirectional, embedded in CIS
	Petersen^23^	2020	Report with risk stratification and recommendations	NR	Risk prediction of neonatal hyperbilirubinemia (jaundice) through bilirubin	Off the shelf, unidirectional data exchange, embedded in CIS
	Thayer^24^	2021	Dashboard	Knowledge	Flag high risk asthma patients	Off the shelf, NR (at least unidirectional), embedded in CIS
3–4 months	Berge^25^	2023	Filtered information in clinical documents	Non-knowledge (NLP)	Identifying and classifying patient allergies	NR, unidirectional data exchange, embedded
	Casey^26^	2023	Risk assessment, report and passive alerts	Non-knowledge-based risk assessment, Knowledge report and passive alerts	Prediction of acute heart failure	Off the shelf, NR, embedded
	Chadwick^27^	2017	Interruptive alerts	Knowledge	Prompt to add HIV test to an order	NR, bidirectional data exchange, embedded in CIS
	Huang^28^	2020	Risk assessment and care bundle	Knowledge	Improve nursing care quality for pressure ulcers	NR, NR (some level of data exchange), NR
	Jenssen^29^	2016	Passive alerts	Knowledge	Smoking cessation counselling and treatment for parents who smoke	Off the shelf, bidirectional data exchange, embedded in CIS
	Keim-Malpass^30^^*,*a^	2018	Risk assessment visualisation	Knowledge	Predictive monitoring and early detection of acute illness	Off the shelf, unidirectional, not embedded in CIS
	Mahabee-Gittens^31^^*,*a^	2018	Passive prompts, interruptive alerts and order set	Knowledge	Tobacco smoke exposure screening and counselling for paediatric caregivers who smoke	Off the shelf, unidirectional data exchange, embedded in CIS
	Rosenthal^32^	2019	Interruptive alerts and order set	Non-knowledge (NLP) triggered alerts and knowledge-based order set	Child physical abuse screening	Off the shelf, unidirectional data exchange, embedded in CIS
	Sauro^19^^*,*a^	2019	*(as above)*			
	Yoon^33^	2023	Imaging detection	Non-knowledge	Detection of 8 abnormal findings in chest x-rays	NR, unidirectional, embedded
4–5 months	Feldstein^34^	2023	Interruptive alerts and order set	Knowledge	Identification, evaluation and reporting of potential child abuse	Off the shelf, bidirectional data exchange, embedded
	Keim-Malpass^30^^*,*a^	2018	*(as above)*			
	Mahabee-Gittens^31^^*,*a^	2018	*(as above)*			
	Suresh^35^	2022	Interruptive alert and screening tool	Knowledge	Child abuse screening to identify child maltreatment	NR, bidirectional, embedded
5–6 months	Ginestra^36^	2019	Interruptive alerts and text message alerts	Non-knowledge	Early warning system to predict severe sepsis or septic shock	NR, unidirectional data exchange, embedded in CIS
	Holroyd-Leduc^37^	2010	Care pathway (strategies; orders; diagnostic tool) embedded in an order set	Knowledge	Delirium prevention among older hip fracture patients	NR, NR, embedded in CIS
	Rabinovich^38^	2022	Imaging detection	Non-knowledge	Detection of pneumothorax, rib fracture, pleural effusion and lung opacities in chest x-rays	NR, NR, embedded
6–7 months	Bellodi^39^	2017	Interruptive alerts (hard and soft stop)	Knowledge	Reduce lab test ordering	NR, bidirectional data exchange, embedded in CIS
	English^40^	2017	Dashboard	NR	Real time surveillance of pharmaceutical therapies	NR, unidirectional data exchange, NR
	Hoekstra^41^	2010	Calculation and recommendations	Knowledge	Potassium regulation, recommendations for pump rate and next administration	NR, unidirectional data exchange, not embedded
	Jones^42^	2019	Interruptive alerts and passive recommendations	Knowledge	ED diagnosis and management of pneumonia	NR, unidirectional data exchange, embedded in CIS
	Uppot^43^	2022	Verbal checklist of targeted electronic health record data	Knowledge	Surgical safety/time-outs performed in ICU	Off the shelf, unidirectional data exchange, not embedded in CIS
7–12 months	Agostini^44^	2008	Interruptive alerts	NR	Educational review and nonpharmacologic alternative recommendation to sedative hypnotic medications for insomnia	NR, unidirectional data exchange, embedded in CIS
	Bell^45^	2019	Interruptive alerts	NR	Antibiotic review, VTE and allergies	Off the shelf, bidirectional data exchange, embedded in CIS
	Cho^46^	2013	Dashboard and data entry form with predictive risk	Non-knowledge (Bayesian Network)	Reducing pressure ulcers	Developed, bidirectional data exchange, embedded in CIS
	Groshaus^47^	2012	Order set	Knowledge	Preventing falls, functional decline and delirium among hospitalised older patients	NR, bidirectional, embedded in CIS
	Jauk^48^	2021	Risk assessment visualisation	Non-knowledge	Delirium prediction	NR, unidirectional data exchange, embedded in CIS
	Lytle^49^	2015	Interruptive alerts	Knowledge	Fall risk identification and prevention: incomplete assessment and high risk of falls/care plan alerts	Off the shelf, bidirectional data exchange, embedded in CIS
	Neame^50^	2021	Interruptive alerts	Knowledge	Medication alerts: dose range checking	Off the shelf, bidirectional data exchange, embedded in CIS
	Nydert^51^	2017	Interruptive alerts	Knowledge	Medication alerts: dose calculation and dose range checking	NR, NR, embedded in CIS
	Pirnejad^52^	2011	Order sets, calculation and recommendations	Knowledge	Chemotherapy protocols and dosing calculations	Developed, NR, embedded in CIS
	Salwei^53^^*,*b^	2021	Passive risk assessment and recommendations	Knowledge	Pulmonary embolism risk assessment and testing	NR, bidirectional data exchange, embedded in CIS
	Salwei^54^^*,*b^	2023	Passive risk assessment and recommendations	Knowledge	Pulmonary embolism risk assessment and testing	Off the shelf, bidirectional, embedded
	Stutman^55^	2007	Interruptive alerts	Knowledge	Medication alerts: drug allergy, drug-drug interactions (critical only), duplicate medication checking and pregnancy and lactation	NR, bidirectional data exchange, embedded in CIS
	Henry^56^	2022	Passive alert and risk assessment	Non-knowledge	Early warning system for timely identification and treatment of sepsis	Off the shelf, bidirectional, embedded
1–2 years	Bersani^57^	2020	Dashboard with passive alerts	Knowledge	Patient safety, across 13 patient safety domains	Developed, bidirectional data exchange, embedded in CIS
	Eden^58^	2020	Interruptive alerts	NR	Medication alerts: allergies and drug interactions	NR, bidirectional data exchange, embedded in CIS
	Frymoyer^59^	2020	Dashboard	Non-knowledge	Precision dosing for vancomycin and therapeutic drug monitoring	Off the shelf, unidirectional data exchange, embedded in CIS
	Goldstein^60^	2022	Interruptive alerts	Knowledge	Identify and refer patients with low vision	Off the shelf, bidirectional, embedded
	Hum^61^	2014	Dashboard and passive recommendations	Knowledge	Improving antibiotic prescribing through recommendations for empiric and targeted therapy	Off the shelf, bidirectional data exchange, embedded in CIS
	Salwei^62^	2022	Passive risk assessment and recommendations	Knowledge	Pulmonary embolism risk assessment and testing	Off the shelf, bidirectional, embedded
	Scheepers-Hoeks^63^	2013	Interruptive and passive alerts	Knowledge	Medication and intervention alerts: 13 clinical rules	Off the shelf, unidirectional data exchange, POC alerts embedded in CIS
	Short^64^	2021	Order with calculations	Knowledge	Increase lung protective ventilation adherence for patients with acute respiratory distress syndrome	Off the shelf, bidirectional data exchange, embedded in CIS
	Zhai^65^	2022	Templates and recommendations	Knowledge	Process-based documentation templates, diagnosis and intervention recommendations	NR, bidirectional, embedded
	Chow^66^^*,*a^	2016	Advice system and alerts	Knowledge	Antibiotic recommendations: type, dose and duration	NR, bidirectional data exchange, embedded in CIS
2–5 years	Campion^67^	2011	Calculation and recommendation	Knowledge	Intensive insulin therapy dose recommendations to maintain blood glucose control	NR, unidirectional data exchange, embedded in CIS
	Chow^68^^*,*b^	2015	Advice system and alerts	Knowledge	Antibiotic recommendations: type, dose and duration	NR, NR, embedded in CIS
	Chow^66^^*,*a,b^	2016	*(as above)*			
	Galanter^69^	2010	Interruptive alerts	Knowledge	IV to oral therapy conversion	Off the shelf, bidirectional data exchange, embedded in CIS
	Lichtner^70^	2020	Powerplan (order set), calculations and interruptive alerts	Knowledge	Chemotherapy prescription and administration, including dosing calculations	Off the shelf, NR, embedded in CIS
	Lin^71^	2010	Indications	Knowledge	Platelet transfusions	NR, NR, embedded in CIS
5+ years	Beeler^72^	2016	Interruptive alerts	Knowledge	Medication alerts: medication allergies, DDIs, duplicate drugs, renal recommendations, age-based recommendations, and formulary substitutions	Developed, bidirectional data exchange, embedded in CIS
	Campion^73^	2011	Calculation and recommendation	Knowledge	Intensive insulin therapy dose recommendations to maintain blood glucose control	NR, unidirectional data exchange, embedded in CIS
	Choi^74^	2019	Interruptive alerts	Knowledge	Renal function drug dosing	NR, bidirectional data exchange, embedded in CIS
	Choudhury^75^^*,*b^	2022	Calculation and recommendations	Non-knowledge	Blood transfusions	NR, unidirectional, embedded in CIS
	Choudhury^76^^*,*b^	2023	Calculation and recommendations	Non-knowledge	Blood transfusions	NR, unidirectional, embedded in CIS
	Luna^77^	2017	Interruptive alerts	NR	Medication alerts: DDIs	Developed, unidirectional data exchange, embedded in CIS
	Ng^78^	2023	Interruptive and passive alerts	Knowledge	Range of best practice advisory alerts	Off the shelf, bidirectional, embedded
	Pontefract^79^	2018	Order sets	NR	Medications (not further reported)	Developed, bidirectional, embedded in CIS
	Van De Sijpe^80^	2022	Passive and interruptive alerts	Knowledge	Screening and alerting module for DDIs	Developed, bidirectional data exchange, embedded in CIS
	Wong^81^	2017	Interruptive alerts	Knowledge	Medication allergy, level 2 DDI alerts, geriatric and renal alerts	Developed, bidirectional data exchange, embedded in CIS
	Wright^82^	2018	Interruptive, passive and hard-stop alerts	Knowledge	DDI alerts	Developed, bidirectional, embedded in CIS

*CDS* clinical decision support, *CIS* clinical information system, *AI* artificial intelligence, *NR* not reported, *VTE* venous thromboembolism, *ICU* Intensive care unit, *HIV* human immunodeficiency virus, *NLP* natural language processing, *ED* emergency department, *IV* intravenous, *DDI* drug-drug interaction.

*CIS details including commercial or developed CIS, level of data exchange between CIS and CDS, CDS embedded or not embedded within CIS, ^a^studies separated for analysis of factors over time, ^b^studies combined for analysis of factors over time.

### Quality assessment

Quality assessment scores for each study are provided in Supplementary Table 5. Using the Mixed Methods Appraisal Tool (MMAT), 32 studies met all 5 quality criteria (48%), 9 met 4 criteria (13%), 10 met 3 criteria (15%), 10 met 2 criteria (15%), 5 met only 1 criterion (7%) and 1 met no criteria (1%). All studies were retained for analysis.

### Time following CDS implementation

CDS were evaluated from 0 months (i.e., immediately following CDS implementation), up to 18 years post-implementation (see Supplementary Table 4). In 5 papers, results were reported within multiple timeframes following CDS implementation due to multiple methods being used or methods being repeated at different time-points. These were included in the review as separate “study time-points”. There were three instances where multiple papers reported findings from the same CDS implementation within the same timeframe following implementation. These six papers were consolidated into three study time-points. This resulted in 70 separate study time-points included in the review. The timeframe containing the highest number of study time-points was the first 6 months following CDS implementation (*n* = 33, 47%).

### Factors influencing acceptance and use over time

A total of 132 unique factors were identified and mapped across six Consolidated Framework for Implementation Research (CFIR) domains (Table 2). Overall, factors within the intervention and inner setting domains were the most frequently reported across studies. Thirty-three unique intervention factors (total *n* = 247; 44% of factors) and 45 unique inner setting factors (total *n* = 164; 29% of factors) were identified. Fewer factors were identified relating to outcomes (*n* = 83, 15%), individuals (*n* = 69, 12%), process (*n* = 21, 4%), and outer setting (*n* = 6, 1%) domains.

**Table 2:** Factors identified by CFIR domains and constructs

				#study timepoints per construct		
CFIR domain	CFIR construct	CFIR sub-construct	Factors	Barriers	Facilitators	Moderators
Intervention	Intervention source		Ownership Locally developed	–	1	–
	Evidence strength and quality		Evidence based Credibility	3	5	–
	Relative advantage		Relative simplicity Usefulness/utility Relative preference Satisfaction Relative efficiency Alert type System performance System quality	17	36	7
	Adaptability		Ongoing adaptation Adaptation speed Personalisation	3	5	–
	Trialability			–	–	–
	Complexity		Simplicity Time and effort Ease of use Cognitive load Ease of learning	11	22	–
	Design quality and packaging		Level of information System feature Visibility of patient status Interface design Additional navigation Device Automaticity Integration Rule or algorithm design Ease of accessing/locating	31	17	–
	Cost			–	–	–
	Data quality^a^		Accuracy of data display Recommendation quality Accuracy of data inputs	10	7	–
Outer setting	Patient needs and resources			–	–	–
	Cosmopolitanism		Site	–	1	4
	Peer pressure			–	–	–
	External policies and incentives		External incentives	–	1	–
Inner setting	Structural characteristics		Transient workforce Governance	2	–	–
	Networks and communications			–	–	–
	Culture		Value to organisation Trust in leadership	–	2	–
	Implementation climate	Tension for change	Adequacy of previous work system Existing practice quality	3	1	–
		Compatibility	Level of duplication Level of manual data entry New work practices Workarounds Alert fatigue Workflow fit Level of interruption Voluntariness End user appropriateness	28	13	–
		Relative priority	Importance of problem Awareness of problem	2	4	–
		Organisational incentives and rewards	Rewards Incentives Expectations	2	–	–
		Goals and feedback		–	–	–
		Learning climate		–	–	–
	Readiness for implementation		Overall readiness Facilitating conditions	1	1	–
		Leadership engagement	Leadership use Early leadership engagement Leadership recommendation	1	3	–
		Available resources	User manual Training Instructions Combination of resources Signage Staffing Technical support	6	10	–
		Access to information and knowledge	Level of clarity around user roles Access to information	2	–	–
	Task and work context^a^		Patient factors Role Time pressure Clinical tasks Medication type Department/unit/ward Existing workload Time of day Shift type Time post CDS trigger Stage of patient journey	13	7	23
Individuals	Knowledge and beliefs about the intervention		Autonomy Usefulness of technology Patient care prioritised Attitude to using Intention to use Risky Trust Would recommend	4	10	–
	Self-efficacy		Understanding and skills Used with clinical judgement Confidence to use Level of reliance (over or under) Used with other sources	12	8	–
	Individual stage of change		Habitual use Ongoing use Personalised use Early impressions	2	9	–
	Individual identification with organisation			–	–	–
	Other personal attributes		(More or Limited) Clinical experience Resilience Individual user differences	2	4	4
Process	Planning			–	–	–
	Engaging		Codesign User engagement Reminders Peer recommendation/support Supervisor recommendation	5	3	–
		Opinion leaders		–	–	–
		Formally appointed internal implementation leaders		–	–	–
		Champions	Champions	3	–	–
		External change agents		–	–	–
	Executing			–	–	–
	Reflecting and evaluating		Iterative approach User feedback System feedback Communication	1	4	–
Outcomes^a^	Innovation deliverers		Prompts consideration Staff communication and coordination Workload Clinician confidence Clinical decision making Efficiency Process complexity Awareness of issue Cognitive load Performance	4	21	–
	Innovation receivers		Patient communication Safety Patient care Patient outcomes Timeliness New errors	10	18	–
	Key decision makers		Guideline adherence Standardisation Culture Productivity Professional development Research	1	7	–

Higher-order factors mapped to the relevant CFIR domain and construct/sub-construct. The number of study time-points reporting barriers, facilitators and moderators in each construct/sub-construct of the CFIR are presented, where constructs were counted once per study time-point (see construct count calculation in Supplementary Table 3).

*CFIR* Consolidated Framework for Implementation Research.

^a^Indicates new domains and/or constructs where factors identified did not align with existing CFIR domains/constructs^100^. Constructs within the ‘outcomes’ domain were informed by the updated CFIR^103^.

**Fig. 2:**
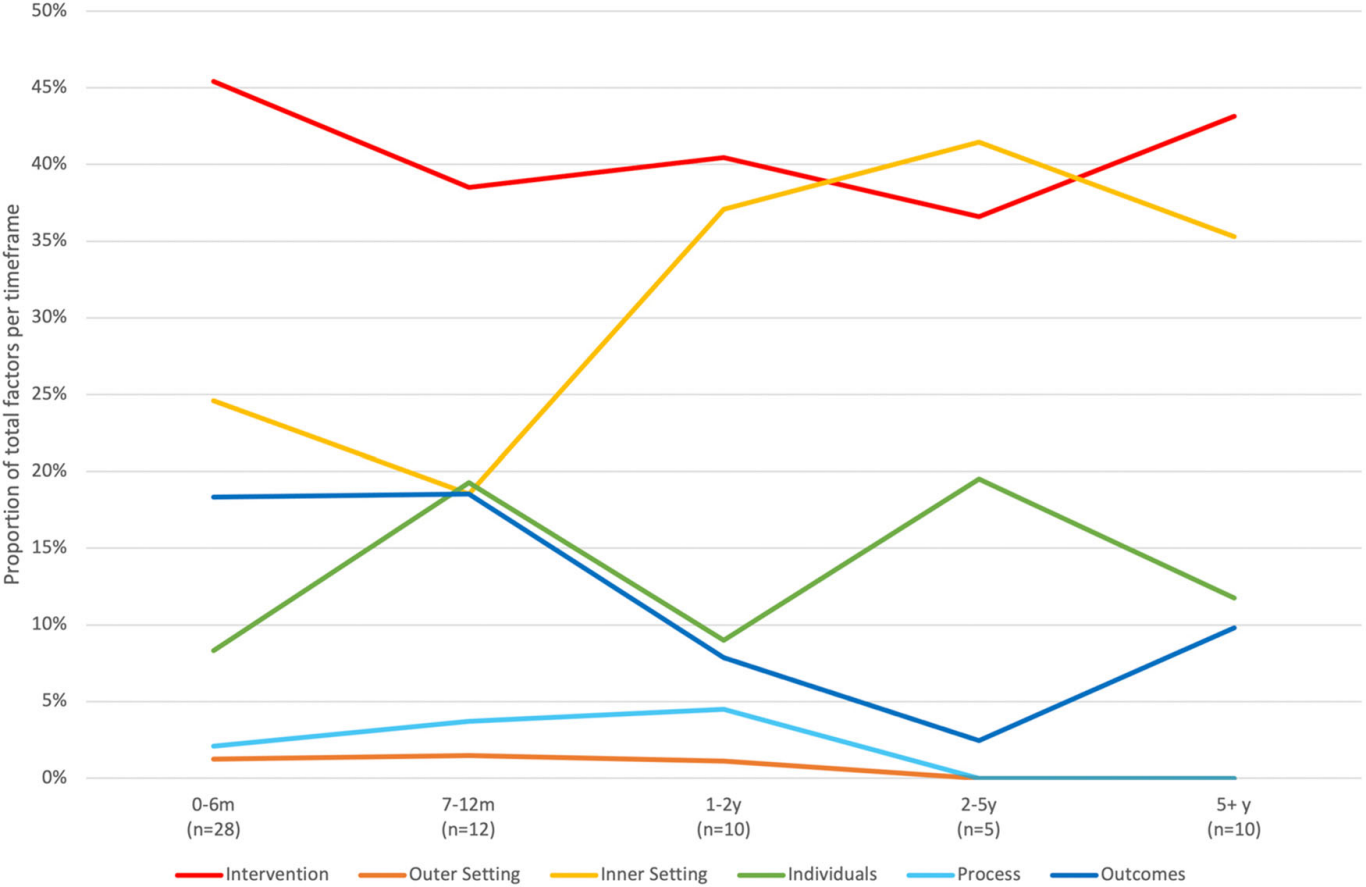
Factors identified in CFIR domains over time. m months, y years, *n* number of study time-points. The proportion of factors identified in each CFIR domain are presented relative to the total number of factors identified within each timeframe. Barriers, facilitators, and moderators were counted once per study time-point (see factor count calculation in Supplementary Table 3) and summed across all study time-points within each timeframe.

As shown in Fig. 2, factors relating to the intervention were prominent in studies conducted at all timeframes following implementation, while those within the inner setting were more often reported after 1 year. Though less frequently reported, factors relating to outcomes (a new domain, not previously in CFIR) were predominantly reported in studies conducted in the first year following CDS implementation, whereas factors relating to individuals were more often reported after 6 months post-implementation.

**Fig. 3:**
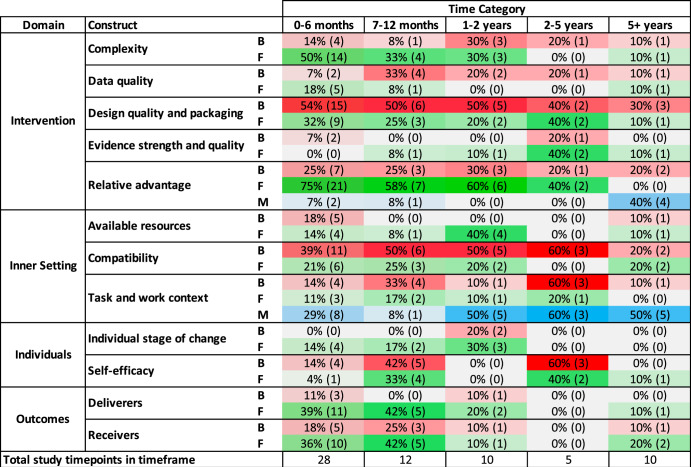
Key constructs identified in CFIR domains over time. Key constructs presented in this figure were identified as barriers, facilitators or moderators in over 25% of study time-points within a given timeframe. The proportion % and number () of study time-points where a construct was identified as a barrier (B), facilitator (F) or moderators (M) to CDS acceptance and use, relative to the total number of study timepoints identified within a given timeframe, are presented. For example, complexity appeared as a barrier in 4 study time-points conducted between 0–6 months, representing 14% of the total 28 study time-points included in this timeframe. Colour saturation was based on the proportion that constructs were reported within each timeframe (i.e. lighter = lower proportion, darker = higher proportion), with red gradients representing barriers, green representing facilitators and blue representing moderating factors. Constructs were counted once per study time-point (see construct count calculation in Supplementary Table 3) and summed across all study time-points within each timeframe.

Figure 3 shows the key constructs reported in each timeframe. Relative advantage (*n* = 60), design quality and packaging (*n* = 48), task and work context (a new construct, not previously in CFIR, *n* = 43) and compatibility (*n* = 41) contained the most barriers, facilitators and moderators reported in study timepoints across timeframes. In Table 3, examples of specific barriers and facilitators identified within these key constructs are presented over time.

**Table 3:** Barriers and facilitators identified in key constructs over time

CFIR domain	CFIR construct	Timeframe				
		0–6 months	7–12 months	1–2 years	2–5 years	5+ years
Intervention	Complexity	• Ease of use (−/+) • Time and effort (−/+) • **Ease of learning (+)**	• Time and effort (−/+) • Ease of use (+)	• Ease of use (+/−) • Time and effort (−) • **Cognitive overload (−)**		
	Data Quality	• Recommendation quality (−/+)	• Recommendation quality (−/+) • Data inputs not trusted (−)	• Recommendation quality (−)		
	Design Quality and Packaging	• Integration of CDS with other systems e.g. EHR (−/+) • Interface design quality (−/+) • Too much/too little information (−) • Valuable system features (+) • **Limited visibility and transparency (−)** • Design of CDS rules or algorithms (−/+)	• Valuable system features/additional features needed (−/+) • Integration of CDS with other systems (−/+) • Too much information (−)	• Integration of CDS with other systems (+) • **Automaticity of CDS (limited use of passive alerts, negative perceptions of interruptive alerts) (−)**		• Additional needs for system features, rules/algorithms, and interface design identified (−)
	Relative Advantage	• Usefulness and utility (−/+) • System performance (−/+) • Preferences for alternate systems (barriers where CDS competed with homegrown CDS and CDS available online; facilitators where CDS was previously paper based) (−/+) • Efficiency over previous system (+) • **Satisfied (+)**	• Usefulness and utility (−/+) • Preferences for alternate systems (−/+) • System performance (−/+)	• Usefulness and utility (−/+) • Efficiency over previous system (+) • Poor system performance (−)	• Usefulness and utility (+)	
	Evidence strength and quality				• **Evidence-based and credible (+)**	
Inner Setting	Available Resources	• Training (−/+) • **Information available (e.g. user manuals, instructions) (−)**		• Training (+)		
	Compatibility	• Workflow fit (−/+) • **Interrupts workflow (−)** • Alert fatigue (−/+)	• Workflow fit (−/+) • Duplication/**less duplication of work (−/+)**	• Workflow fit (−/+)	• Duplication of work (−)	• **Workarounds (−/+)** • Alert fatigue and reductions in alert fatigue following modifications (−/+)
	Task and Work Context	• Time pressure and existing workload (−) • Useful or not useful for specific clinical tasks or patients (−/+)	• Time pressure and existing workload (−) • Useful or not useful for specific clinical tasks (−/+)		• Useful or not useful for specific clinical tasks or patients (e.g. complex patients) (−)	
Individuals	Individual stage of change			• **Early impressions of CDS (−)**		
	Self-Efficacy	• Lack of understanding and skills to use CDS (−)	• Lack of vs. **sufficient understanding and skills to use CDS** (−/+) • **Potential for over reliance (−)** • CDS used alongside clinical judgement (−/+)		• CDS used alongside clinical judgement and prior experience (−/+)	
Outcomes	Innovation Deliverers	• Improved staff communication and collaboration (+) • **Improved/impaired clinical decision making (−/+)** • Prompted consideration (+) • **Enhanced confidence (+)** • Increased/**reduced efficiency** (−/+)	• **Reduced/did not reduce workload (−/+)** Prompted consideration (+) Improved staff communication and collaboration (+) Increased efficiency (+)			
	Innovation Receivers	• Improved/**did not change patient care** (−/+) Improved/reduced patient safety (−/+) • **Increased patient communication (+)** • **Improved/did not change patient outcomes (−/+)** • **More timely care (+)**	• Improved (e.g. reduced errors) /reduced patient safety (incl. **new system-related errors**) (−/+) • **Delays in care (−)** • Improved patient care (+)			

Key constructs presented in this table were identified as barriers or facilitators in over 25% of study time-points within a given timeframe. Factors reported were identified in 2 or more studies within each timeframe where (+) indicates a facilitator to acceptance and use i.e. positive direction, and (−) indicates a barrier to acceptance and use i.e. negative direction. Factors in **bold** were uniquely reported within a particular timeframe.

*CFIR* Consolidated Framework for Implementation Research*, EHR* Electronic Health Record, *CDS* clinical decision support.

#### 0–6 months following CDS implementation

Twenty-eight studies were conducted in the first 6 months following CDS implementation^16–43^. Four of these studies^16,19,30,31^ evaluated acceptance and/or use at multiple months post-implementation, yielding 33 study time-points within this timeframe (see Table 1). Factors identified (*n* = 245) most often related to the intervention, followed by the inner setting, outcomes, individuals, process and outer setting, as shown in Fig. 2. Supplementary Fig. 1A, B shows the proportion of barriers and facilitators identified in each domain over monthly intervals. Both barriers and facilitators in the intervention domain remained most frequently reported at most monthly intervals, though those relating to outcomes trended upward across this timeframe.

**Fig. 4:**
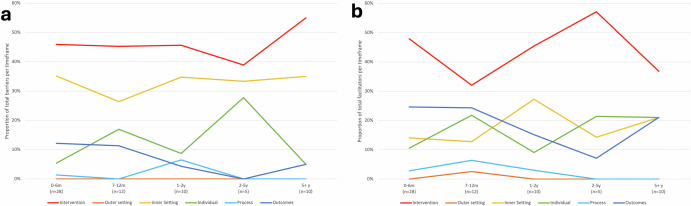
Barriers and facilitators identified in CFIR domains over time. m months, y years, *n* number of study time-points. **a** The proportion of *barriers* identified in each CFIR domain are presented relative to the total number of barriers identified within each timeframe. Barriers were counted once per study time-point (see factor count calculation in Supplementary Table 3) and summed across all study timepoints within each timeframe. **b** The proportion of *facilitators* identified in each CFIR domain are presented relative to the total number of facilitators identified within each timeframe. Facilitators were counted once per study time-point (see factor count calculation in Supplementary Table 3) and summed across all study timepoints within each timeframe.

The proportion of barriers and facilitators identified in each domain across all timeframes are presented in Fig. 4a, b. In the first 6 months post-implementation, barriers (*n* = 80) within the intervention and inner setting domains were more frequent relative to other domains (Supplementary Fig. 1A). In contrast, facilitators (*n* = 146) within the intervention and outcomes domains were more often identified (Supplementary Fig. 1B). Examples of common barriers and facilitators identified during the first 6 months following CDS implementation are presented in Supplementary Table 6.

#### 7–12 months following CDS implementation

Thirteen studies, evaluating 12 unique systems, were conducted between 7–12 months following implementation, resulting in 12 study time-points (see Table 1)^44–56^. Factors (*n* = 137) relating to the intervention remained most prevalent, despite decreasing from the previous timeframe. Those relating to individuals increased, whereas those in the inner setting domain decreased and those in outcomes, process and outer setting domains remained relatively stable (Fig. 2).

Barriers (*n* = 55) were most frequently identified within intervention and inner setting domains, though those relating to individuals and outcomes were also prevalent in studies conducted during this time (Fig. 4a). Despite decreasing in incidence from 0–6 months post-implementation, facilitators (*n* = 78) in the intervention domain remained the most reported (Fig. 4b). Facilitators relating to outcomes and individuals were also frequently reported.

#### 1–2 years following CDS implementation

Ten studies were conducted between 1–2 years following CDS implementation^57–66^. Factors (identified *n* = 90) remained most frequent in the intervention domain, closely followed by the inner setting, increasing from the previous timeframe. Those reported in individuals and outcomes domains decreased, whereas those in process and outer setting domains remained low (Fig. 2).

Barriers (*n* = 46) within the intervention and inner setting domains remained the most frequently identified during this timeframe, followed by those in the individuals domain (Fig. 4a). Facilitators (*n* = 33) identified in the intervention and inner setting domains were most prevalent, both increasing in incidence from the previous timeframe, while those relating to outcomes and individuals decreased (Fig. 4b).

#### 2–5 years following CDS implementation

Six studies were conducted between 2–5 years following CDS implementation, with 5 study time-points identified (see Table 1)^66–71^. One study^66^ reported factors between 1–2 years, and between 2–5 years. Factors (*n* = 41) within the inner setting domain slightly increased, followed by those within the intervention domain which slightly decreased (Fig. 2). Within the individuals domain, the incidence of factors increased, whereas those within the outcomes domain decreased. There were no factors identified within process or outer setting domains.

Barriers (*n* = 18) were most frequently identified within the intervention domain and inner setting despite decreasing from the previous timeframe, while the incidence of barriers in the individuals domain tripled (Fig. 4a). Facilitators (*n* = 14) within the intervention and individuals domains were most frequently identified, both increasing in incidence during this timeframe (Fig. 4b). Those relating to the inner setting and outcomes domains however, decreased.

#### Over 5 years following CDS implementation

Eleven studies were conducted 5 or more years following CDS implementation, with 10 study time-points identified (see Table 1)^72–82^. Factors (*n* = 51) relating to the intervention were most frequently reported, rising from the previous timeframe (Fig. 2). This was followed by factors relating to the inner setting, and individuals, which decreased from the previous timeframe, and those relating to outcomes, which increased. Consistent with the previous timeframe, no factors relating to the process or outer setting were identified.

Barriers (*n* = 20) within the intervention domain were most frequently identified during this timeframe, followed by those related to the inner setting. Those relating to individuals and outcomes domains were low (Fig. 4a). Facilitators (*n* = 19) were most frequently identified in the intervention domain, decreasing from the previous timeframe. This was followed by the inner setting and individuals, remaining relatively stable compared to the previous timeframe, and outcomes, which increased (Fig. 4b).

#### Moderating factors influencing CDS acceptance and use

Moderating factors (*n* = 55) were more often identified in studies conducted over a year following CDS implementation (between 50–60% of studies conducted during this time) (Fig. 3). Moderators were primarily identified in the ‘task and work context’, including the clinical user role e.g. nurses typically held more favourable views than doctors^18,36^ and junior doctors typically held more favourable views than senior doctors^68^. The department, unit or ward, the patient/population and type of shift where CDS was used e.g. whether clinicians were on call, also influenced acceptance and use (Table 2).

## Discussion

We conducted a systematic review of studies that reported factors influencing clinicians’ acceptance and use of CDS systems following their implementation in hospital settings. Our findings align with previous reviews of studies evaluating clinicians’ experiences of CDS, which highlight technological and organisational factors, such as usefulness, usability and fit with workflows, as key issues^6–10^. To our knowledge, this is the first review to collate and synthesise factors according to the point in time they were reported post-implementation. In doing so, we expand on previous work by identifying important themes relating to clinicians’ acceptance and use of CDS over time. We discuss these themes in the context of existing research and outline their implications for design, implementation, and evaluation of CDS systems, and reporting of future research.

Firstly, our synthesis suggests that certain barriers can arise early post-implementation and may continue to be experienced if not actively addressed at an early stage^83^. We found barriers relating to the system and inner setting, including poor design quality and packaging, relative advantage, and compatibility between the CDS and existing workflows, were frequently reported across all timeframes (Figs. 3 and 4a). Specific barriers within these constructs, such as poor integration and interoperability between CDS and the EMR, fit with workflows, and system performance, were reported in studies conducted early after implementation and up to 2 years post (Table 3). Identifying and addressing these barriers soon after they emerge may improve the likelihood of uptake and sustained use.

Some barriers may be experienced more prominently in the immediate period following CDS implementation and resolve as clinicians become increasingly familiar with the system, as the system is adapted to meet local needs, and as clinicians develop strategies to overcome system limitations over time^84^. We found issues in the intervention, inner setting, and outcomes domains, such as limited transparency of CDS, a lack of resources to support CDS use, and reduced efficiency, to be primary concerns in the first 6 months post-implementation (Table 3). However, in the following timeframe (7–12 months), there was an increase in facilitators, and decrease in barriers, relating to clinicians’ skills to use CDS appropriately. Additionally, workarounds to overcome system limitations were rarely identified until later phases of use. These findings align with existing research evaluating computerised provider order entry (CPOE) systems, where clinicians’ inexperience and unfamiliarity contributed to an increase in barriers and errors reported in the immediate post-implementation period^12,85^. Our review, like other studies^12,86^, suggests users develop ways to work around system limitations over time. Though workarounds can be positive, allowing for users to overcome design and workflow inefficiencies, they may also increase the risk of errors occurring^86^. Thus, enhancing CDS design early may minimise workarounds^87^, and providing clinicians with enhanced support, such as ongoing training and information sessions, may be helpful to overcome challenges associated with a lack of familiarity during the early phases of use^56^.

Our review also suggests that clinicians’ ability to recognise certain barriers and outcomes increases over time as they become more experienced with CDS. We found concerns about the accuracy of data inputs driving CDS recommendations to be rarely identified until 7–12 months post-implementation, coinciding with an increase in users’ understanding of the system. For example, in Lichtner et al.^70^, clinicians became more ‘watchful’ of automated behaviour with increased use. Similarly, the prevalence of outcomes identified in our review increased over the first 6 months following implementation (Supplementary Fig. 1A, B) and up to 1-year post-implementation (Figs. 2 and 3). We found some negative outcomes, including delays in care and new system-related errors to be reported only in studies conducted between 7–12 months after implementation. These findings echo existing research evaluating CPOE systems over time^12,84^.

Additionally, changes to the work system and associated context may impact issues experienced over time. In Salwei et al.^53^ and Campion et al.^73^, changes made to related clinical information systems (CIS) resulted in the disruption of CDS workflows at 1 year, and over 5 years, following CDS implementation. Building on recommendations from previous reviews, these findings exemplify the importance of engaging clinicians not only during CDS development, but on an ongoing basis to identify and address both expected and unanticipated issues that may arise over time^6^. Furthermore, engaging users prior to changing existing, or deploying new, CIS systems may help to uncover potential workflow impacts to systems already in use. Despite this, no factors related to the implementation process were identified beyond 2 years post-implementation, indicating that strategies such as user feedback and system monitoring to address persistent or late-emerging barriers are rarely utilised long-term.

Staffing changes, such as the rotation of clinicians, and new users of CDS are inevitable and likely to impact how CDS is accepted and used over time^13^. However, only one study reported the impact of the organisations’ transitory workforce^53^ and no studies investigated how new users adopted existing CDS. The lack of factors relating to available resources identified in later timeframes suggests limited training and education opportunities to support later adopters. Similarly, there were very few studies that reported factors relating to the outer setting environment, with none discussing regulatory or clinical guideline changes that could affect CDS acceptance and use over time. Such topics warrant future research.

Results showed that outcomes may become less visible to clinicians over a prolonged period of time. Interestingly, both positive and negative outcomes were rarely identified in papers beyond 1-year post-implementation (Fig. 2). Such findings could reflect the process of ‘normalisation’, in which a system becomes increasingly integrated into routine practice and consequently ‘disappears from view’^15^. Changes to the CDS system however, may spark new benefits realisation. A slight peak in the prevalence of positive outcomes reported in studies conducted beyond 5 years after implementation coincided with intervention factors commonly reported during this time, such as ongoing design needs and modifications (Fig. 4b and Table 3). Our findings suggest that those looking to evaluate perceived benefits of CDS systems, should do so within the first year following implementation, before CDS becomes normalised. Future studies should also explicitly explore how perceived outcomes of CDS change over time and whether and how perceived CDS outcomes are sustained, given the lack of outcomes identified in later phases of use may reflect a lack of long-term benefits evaluation in existing studies.

Lastly, clinicians appeared to be able to better understand advantages and limitations of CDS with increased use. Factors relating to clinicians’ self-efficacy to use CDS increased over time, with studies conducted between 2–5 years post-implementation often reporting that clinicians combined their clinical judgement, intuition and experience with CDS recommendations, and rejected recommendations where CDS did not align (Fig. 3 and Table 3)^67,68,70^. This finding is particularly interesting, given increasing concerns of automation bias leading to over-reliance on CDS^88^. While a few studies reported that clinicians were concerned about the ‘potential’ for over-reliance on CDS^21,51,56^, findings from the review support the theory that clinicians can more accurately consider limitations with increased use of and familiarity with the system over time^88^.

Almost half the studies included in this review were conducted during the first 6 months following implementation (28/67) and fewer factors were identified in studies conducted in later timeframes, particularly >2 years post-implementation (92/556 factors). This indicates a need for further research to comprehensively explore the factors driving sustainable use of CDS systems. Furthermore, there were limited studies that evaluated acceptance and use of CDS at different points in time within a single study. Studies that reported findings at multiple points in time, did so as a consequence of employing multiple methods of evaluation, rather than purposefully exploring changes over time. Though a few studies identified in our search explored clinicians’ acceptance of CDS over time, these studies did not provide the point-in-time that CDS systems were evaluated^11^ or reported findings on a broader CIS implementation (i.e. did not report findings related specifically to CDS systems)^12,84,89^, and thus were excluded from this review.

Though the CFIR provided a useful lens to systematically consider factors related to CDS acceptance and use, we identified additional factors that did not fit within the existing framework. These included the quality of data inputs and outputs of the CDS system in the *intervention* domain, the task and work context in the *inner setting* domain, and perceived outcomes, which should be considered when evaluating acceptance and use of CDS systems. Importantly, while our review suggests that factors influencing clinicians’ acceptance and use CDS systems can change over time, 196 papers that would have otherwise been included did not report the time following implementation that evaluation was completed and were excluded from the review. We therefore urge future studies examining acceptance and use of CDS and other digital health interventions to report the time of data collection in relation to implementation. We also recommend that research reporting guidelines be updated to make reporting of time between implementation and evaluation of interventions a requirement.

A key limitation of this review was the between-studies design and heterogeneity of included studies. Thus, factors identified within timeframes may have been influenced by differences in CDS systems, users, settings, methods used, and specific focus of studies conducted at each point in time. Further research employing longitudinal, within studies designs are required to confirm and expand upon the findings laid out in this review. Additionally, as the majority of included studies were conducted in the US and other high-income countries, findings may have limited generalisability to other settings, particularly developing countries.

While we only included studies that specified the time of data collection following implementation, ‘implementation’ may have been interpreted and reported inconsistently between studies. For example, CDS systems may be implemented in a limited capacity before full implementation whereas others may be implemented using a ‘big bang’ approach^90^. Likewise, CDS systems are often updated and adapted following their initial ‘go-live’ date^89^, but this detail is rarely provided in publications. This indicates a need for future research that evaluates what and how changes, such as adaptations to CDS and new users, can impact clinicians’ acceptance and use of CDS over time.

Our review provides practical guidance to assist stakeholders in anticipating and identifying issues likely to impact CDS acceptance and use over time. We emphasise the importance of engaging clinicians early after implementation, and on an ongoing basis, to ensure issues that develop over time are promptly and successfully addressed. We must move away from episodic evaluations of clinicians’ acceptance and use of CDS systems and towards a framework that considers the complexity of factors, including how they emerge, interact, and change over time. Doing so will allow for more efficient and nuanced approaches that target the issues clinicians experience at different points in time, increasing the likelihood of sustained system success. Reporting the time of data collection post-implementation and employing longitudinal designs in future research is necessary to achieve this goal.

## Methods

This systematic review is reported following the Preferred Reporting Items for Systematic Reviews and Meta-Analyses (PRISMA) guidelines^91^. The protocol for this review is registered in PROSPERO (CRD42022325469). Two related reviews, each with minor variations in study inclusion criteria, have been previously published as conference papers. These papers aimed to review the methods used to evaluate clinicians’ acceptance and use of CDS over time^92^, and the use of approaches to involve clinicians in CDS design on post-implementation acceptance and use^93^, which differed from the aims of the current review.

### Search strategy

Ovid MEDLINE, Embase, Web of Science, CINAHL and PsycINFO were systematically searched on 17 March 2022, with an additional search conducted on 19 January 2024, to identify studies reporting clinicians’ acceptance and use of CDS following implementation in hospital settings. Our search was restricted to studies published within 15 years of the initial search date (i.e. from January 2007) to ensure studies reflected current CDS systems and organisational environments. A professional librarian was consulted in the development of the search strategy (see Supplementary Note 1). A manual search of reference lists of relevant reviews was also conducted.

### Inclusion and exclusion criteria

CDS systems were defined as electronic systems that aim to enhance clinical decisions with targeted clinical knowledge and patient information to support individual patient care^94^. All types of CDS systems (e.g., alerts, dashboards) were considered in scope, however eligible CDS must have been integrated with a CIS (e.g., Electronic Medical or Health Records, and Computerised Provider Order Entry systems). Our population of interest included any hospital-based clinicians (e.g. doctors, nurses) who were end users of a CDS system, targeting any health condition or patient group. Eligible studies reported factors influencing clinicians’ perceptions of (acceptance), and/or actual interactions (use) with a CDS system to support patient care in inpatient or outpatient hospital settings. Studies that evaluated CDS as part of a broader system were only included if CDS-specific results were reported. We included peer-reviewed original research and case studies that employed qualitative, quantitative or mixed-methods designs, and were available in English. To capture the point-in-time that factors emerged, eligible studies needed to report the specific timing of data collection in relation to CDS implementation.

### Study selection

After removing duplicates using EndNote 20 software^95^, titles and abstracts were imported into Covidence (www.covidence.org) and independently screened for inclusion by two authors (NN and AB, RF or MB). Full texts of potentially relevant articles were screened against inclusion criteria. A sample of full texts were independently screened by review pairs (NN and AB, RF or MB), until Cohen’s kappa of >0.81, representing ‘almost perfect’ interrater reliability, was achieved^96^. Disagreements were resolved through discussion between the review pair and if required, discussion and consensus among four authors. Remaining texts were screened by one reviewer each (NN, AB, RF or MB).

### Data extraction

Data were extracted independently by two authors (NN and AB, RF, AT or MB) using a structured data collection form in Microsoft Excel. The form was developed and iteratively refined following extraction of data from a sample of studies. Data extracted included study details and identifiers (e.g. authors, year), participant role, setting (e.g. department, unit), CDS description (e.g. type, AI vs. non-AI based), acceptance and/or use measurement, factors associated with clinicians’ acceptance and/or use of CDS, and the time of data collection following implementation. Missing or unclear information were recorded as not reported (NR). Disagreements were resolved as described above.

### Quality assessment

The methodological quality of included studies was independently appraised by two authors (NN and AB, RF, AT or MB) using the well-established MMAT^97^. Quality was assessed only for study methods and results that met inclusion criteria (see Supplementary Note 3). Disagreements were resolved as described above. As we aimed to comprehensively identify the factors observed to influence CDS acceptance and use over time, quality assessment scores were not used as a basis for exclusion of studies but to guide the interpretation of findings^98,99^.

### Data analysis and synthesis of factors over time

We used the CFIR to synthesise findings^100^. The CFIR is an implementation science framework that is widely used in healthcare settings to evaluate individual, technological and contextual factors influencing the implementation of complex interventions. The CFIR was selected for this review because of its comprehensive structure, ensuring factors were captured via a whole of system approach. Factors were synthesised using a convergent integrative approach^101^, where a word or short phrase that captured the meaning and direction (i.e. positive, negative, no direction) of factors reported in studies was noted under one of five major domains in the CFIR framework: intervention, outer setting, inner setting, individuals, and process.

Following extraction of all data, five reviewers (NN, AB, RF, MB, AT) participated in 7 workshops totalling 11 h to synthesise factors under CFIR constructs. This included discussion of each original factor documented in the data extraction form and in some cases, merging and/or renaming factors, before allocating to constructs. This created a framework of CFIR domains, constructs and factors that influenced CDS acceptance and use. In cases where factors did not align with any existing CFIR constructs or domains, new constructs or domains were created. All factors were classified as barriers, facilitators or moderators. Moderating factors were those that influenced the level of CDS acceptance and/or use.

The point-in-time that data were collected in each study was grouped into a time category (‘timeframe’) in line with inclusion criteria detailing time specificity that is described in Supplementary Note 2 and Supplementary Table 1. Graphs were created in Microsoft Excel and Tableau^102^ to visualise the occurrence and proportion of overall factors, constructs, barriers, facilitators reported in different domains at each timeframe.

## Supplementary information

Supplementary materials

## Data Availability

The datasets analysed during the current study are available from the corresponding author upon request.
