# Supplementary material for: A systematic review of clinicians’ acceptance and use of clinical decision support systems over time
Source: NPJ Digit Med. 2025 May 26;8:309. doi: 10.1038/s41746-025-01662-7 (PMC12106704; doi:10.1038/s41746-025-01662-7)
Supplement: Supplementary file 1 — Supplementary materials [file 41746_2025_1662_MOESM1_ESM.pdf]

Factors influencing clinicians' acceptance and use of clinical decision support systems over time: A systematic review

Supplementary Materials File

Table of Contents

Supplementary Note 1. Search terms 2

Supplementary Note 2. Time specificity inclusion criteria and reporting 5

Supplementary Table 1. Time specificity criteria description and examples 5

Supplementary Table 2. Common examples for excluding study results 6

Supplementary Table 3. Denominator definitions and working example 7

Supplementary Table 4. Study methods, timeframe and characteristics 8

Supplementary Note 3. Detailed quality appraisal results 22

Supplementary Table 5. Quality appraisal using the MMAT 22

Supplementary Figure 1. Barriers and facilitators identified in CFIR domains 1-6 months following CDS implementation 26

Supplementary Table 6. Barriers and facilitators identified in key constructs 1-6 months following CDS implementation 27

Supplementary Table 7. Completed PRISMA 2020 Checklist 29

Supplementary Note 1. Search terms

Searches were conducted on the following dates:

- Initial search: 17 March 2022
- Updated search: 19 January 2024

Databases: Medline, Embase, Web of Science, CINAHL and PsycINFO

Medline

(exp decision support systems, clinical/ or "decision support system*".tw. or "clinical decision support*".tw. or exp clinical decision rules/ or "computeri*ed decision support*".tw.) and (Accept* or adopt* or attitude* or percei* or percep* or barrier* or facilitator* or useful* or usab* or Uptake or perspective* or satisf* or concern* or (observation* or interview* or survey* or questionnaire* or "focus group*")).tw. and (exp hospitals/ or exp hospital units/ or exp hospital departments/ or hospital*.tw. or outpatient*.tw. or inpatient*.tw.)

Embase

(exp "Clinical decision support system"/ or "decision support system*".tw. or "clinical decision support*".tw. or "computeri*ed decision support*".tw. or exp "clinical decision rule"/) and (Accept* or adopt* or attitude* or percei* or percep* or barrier* or facilitator* or useful* or usab* or Uptake or perspective* or satisf* or concern* or usage or "system use" or (observation* or interview* or survey* or questionnaire* or "focus group*" or "log data")).tw. and (exp "hospital"/ or exp "hospital subdivisions and components"/ or exp "hospital department"/ or hospital*.tw. or outpatient*.tw. or inpatient*.tw.)

Web of Science

(TI=("clinical decision support*"
"decision support system*" OR
"computeri*ed decision support*" OR
"clinical decision support*" OR
"clinical decision rule*") OR AB=("clinical decision support*"
"decision support system*" OR
"computeri*ed decision support*" OR
"clinical decision support*" OR
"clinical decision rule*")) AND
(TI=((Accept* OR
adopt* OR
attitude* OR
percei* OR
percep* OR
barrier* OR
facilitat* OR
useful* OR
usab* OR
Uptake OR

perspective* OR
satisf* OR
concern* OR
usage OR
"system use") OR
(observation* OR
interview* OR
survey* OR
questionnaire* OR
"focus group*"OR
"log data")) OR AB=((Accept* OR
adopt* OR
attitude* OR
percei* OR
percep* OR
barrier* OR
facilitat* OR
useful* OR
usab* OR
Uptake OR
perspective* OR
satisf* OR
concern* OR
usage OR
"system use") OR
(observation* OR
interview* OR
survey* OR
questionnaire* OR
"focus group*" OR
"log data"))) AND
(TI=(Hospital* OR
inpatient* OR
outpatient* OR
"hospital department*" OR
"hospital unit*") OR AB=(Hospital* OR
inpatient* OR
outpatient* OR
"hospital department*" OR
"hospital unit*"))

CINAHL

(MH "Decision Support Systems, Clinical+" OR TI "decision support system*" OR AB "decision support system*" OR TI "computeri*ed decision support*" OR AB "computeri*ed decision support*" OR TI "Clinical decision support*" OR AB "Clinical decision support*" OR MH "Clinical prediction rules+") AND ((MH "Attitude of Health Personnel+" or ti Accept* or ab accept* OR ti

adopt* OR ab adopt* or ti
attitude* OR ab attitude* or ti
percei* OR ab percei* or ti
percep* OR ab percep* or ti
barrier* OR ab barrier* or ti
facilitator* OR ab facilitator* or ti
useful* OR ab useful* or ti
usab* OR ab usab* or ti
Uptake OR ab uptake or ti
perspective* OR ab perspective* or ti
satisf* OR ab satisf* or ti concern* or ab concern* OR ti usage or ab usage or ti "system use" or ab "system use") OR (ti
observation* OR ab observation* or ti
interview* OR ab interview* or ti
survey* OR ab survey* or ti
questionnaire* OR ab questionnaire* or ti
"focus group*" or ab "focus group*" or ti
"log data" or ab "log data")) AND (MH Hospitals+ OR TI hospital* or ab hospital* or ti inpatient* or ab inpatient* OR ti
outpatient* OR ab outpatient* or MH
"hospital units+" or ti "hospital department*" or ab "hospital department*")

PsycINFO
(exp decision support systems/ or "decision support system*".tw. or "clinical decision support*".tw. or "computeri*ed decision support*".tw. or clinical decision rule*.tw.) and (exp health personnel attitudes/ or accept*.tw. or adopt*.tw. or attitude*.tw. or percei*.tw. or percep*.tw. or barrier*.tw. or facilitator*.tw. or useful*.tw. or usab*.tw. or Uptake.tw. or perspective*.tw. or satisf*.tw. or concern*.tw. or usage.tw. or "system use".tw. or (observation* or interview* or survey* or questionnaire* or "focus group*" or "log data").tw.) and (exp hospitals/ or "hospital unit*".tw. or "hospital department*".tw. or hospital*.tw. or outpatient*.tw. or inpatient*.tw.)

Supplementary Note 2. Time specificity inclusion criteria and reporting

Time specificity

As the early phases of implementation are often associated with fast-paced changes in perceptions, attitudes, and use,[11] we required time to be reported at a greater level of detail the closer evaluations were conducted to CDS implementation. Supplementary Table 1 shows the level of time specificity that was required for study inclusion, between different timeframes.

In papers that reported results at multiple time points following CDS implementation, factors were extracted on separate rows of the data extraction form and categorised into separate 'study time-points' for the purposes of the review. Similarly, where multiple papers described the same CDS implementation at the same point in time following implementation, factors were combined into a single row and categorised as whole study time-points for the purposes of the review.

Supplementary Table 1. Time specificity criteria description and examples

	**Time following implementation**			
	**0-6 months**	**7-24 months**	**2-5 years**	**5+ years**
Reporting required	Monthly level	6-monthly level	Any time between interval	Any time
Description	The month and year of data collection and month and year of CDS implementation reported OR the time (in months) that the study was conducted following CDS implementation was reported	Time of data collection following implementation was reported at 6-month specificity	Time of data collection following implementation was reported anywhere between 2-5 years following implementation	Time of data collection following implementation reported at any time over 5 years
Example	CDS was implemented in June 2015 and data were collected in July 2015	CDS was implemented in June 2015 and data were collected between February 2015 and May 2015	CDS was implemented in 2015 and data were collected in 2018	CDS was implemented over 5 years prior to data collection

Supplementary Table 2. Common examples for excluding study results

Combination	Example issue	Outcome
Multiple data collection methods/timeframe	Time of questionnaire following implementation was reported but timeframe of interviews was not	Questionnaire results included and extracted; interview results excluded
Multiple CDS/timeframe	Time of data collection following implementation of one CDS reported, but another CDS not	CDS with time reported included, CDS without time reported excluded
Multiple sites/timeframe	Time of data collection following implementation of CDS in one site reported, but another site not	Site with time reported included, site without time reported excluded
Multiple CDS/CIS integration	One CDS was integrated with the CIS and another was not	CDS integrated with the CIS included, CDS not integrated with CIS excluded
Multiple implementations/ settings	CDS was implemented in both hospital and primary care settings	CDS implemented in hospital included, primary care excluded
Multiple methods/stage of implementation	Questionnaire conducted pre-implementation and post-implementation	Post implementation findings included, pre implementation findings excluded

Supplementary Table 3. Denominator definitions and working example

Working example<br>In Salwei et al.[52], within the 'design quality and packaging' construct, we identified 1 facilitator and 2 barriers to 'system features', 1 barrier and 1 facilitator to 'integration', and 1 facilitator to 'ease of accessing/locating'		
**Denominator**	**Definition**	**Example**
Factor count	Factors within a construct were counted per study timepoint, per direction of factor	Factors within the 'design quality and packaging' construct were identified as facilitators 3 times (*1x system features, 1x integration, 1x ease of accessing/locating),* and as barriers 2 times *(1x system features, 1x integration*)
Construct count	Constructs were counted once per study timepoint, per direction of construct	The 'design quality and packaging' construct was identified as a facilitator once (*1x design quality and packaging*) and a barrier once (*1x design quality and packaging*)

Supplementary Table 4. Study methods, timeframe and characteristics

Time	First author	Year	Country	Setting	Participants	Partial or full study	Study design	Data source	Use measurement	Acceptance measurement	Data collection timeframe
0-1 months	Castellanos[16,a]	2018	Germany	Tertiary care university hospital, surgical ICU	Physicians	Partial	Longitudinal	System data	Individual tests for which the CDS was consulted and adherence to recommendations (event level)	-	0-28 days
	Grauer[17]	2022	USA	11 hospitals and 200 clinics, inpatient and outpatient excluding ED and urgent care	Physicians and registered nurses	Partial	Cross sectional	System data	Alert acceptance (event level)	-	0-5 weeks
1-2 months	Castellanos[16,a]	2018	Germany	*(as above)*							28-56 days
	Guidi[18]	2015	USA	Academic health system, hospital wide	Physicians, advanced practice providers and registered nurses	Full	Cross sectional	Survey	-	Utility of alerts	1-2.5 months

	Sauro[19,a]	2019	Canada	Healthcare system, 4 adult medical-surgical ICUs	Physicians, nurse practitioners, medical trainees (residents and clinical fellows), nurses, and pharmacists	Full	Cross sectional	Survey	-	Acceptability, adoption, appropriateness, feasibility, and penetration of CDS	1 month
	Tsai[20]	2022	Taiwan	3 hospitals, EDs	Physicians and nurses	Partial	Cross sectional	Survey	-	TAM survey: Perceived ease of use, perceived usefulness, and perceived acceptance	1 month
2-3 months	Castellanos[16,a]	2018	Germany	*(as above)*				Interviews	-	Qualitative	2-3 months
	DeBie[21]	2021	Netherlands	Tertiary hospital, mixed medical and surgical ICU	Intensivists, residents, ICU physician assistants	Partial	Pre-post	Interviews, questionnaire	-	Usability questionnaire: pragmatic quality, hedonic quality – identity, hedonic quality – stimulation, attractiveness, and user acceptance questionnaire based on TAM	2.5 months
	Harrison[22]	2017	USA	Hospital, medical ICU	Nurse practitioners, physician assistants, physicians	Partial	Cross sectional	Survey, system data	Alert acceptance (event level and clinician level)	User satisfaction	2 months

	Petersen[23]	2020	USA	2 hospitals, newborn nurseries	Neonatal nurse practitioners, paediatric resident physicians, neonatology fellows, paediatric hospitalists, and neonatologists	Partial	Pre post	Survey	Self-reported use	User experience	2 months
	Thayer[24]	2021	USA	Large academic tertiary care children's hospital, emergency department	Physicians	Partial	Cross sectional	Observations, interviews	-	Qualitative	2 months
3-4 months	Berge[25]	2023	Norway	Hospital, anaesthesia and ICU department	Doctors and nurses	Partial	Cross sectional	Survey	-	UTAUT survey: Performance expectancy, effort expectancy, facilitating conditions, social influence, intention to use the system	3 months
	Casey[26]	2023	USA	2 hospitals, EDs	Physicians	Full	Cross sectional	Interviews, survey	Self-reported use	Qualitative and usability testing	3 months
	Chadwick[27]	2017	UK	Urban hospital, hospital wide	Doctors and nurse practitioners	Partial	Cross sectional	Interviews, focus groups	-	Qualitative	3 months
	Huang[28]	2020	Taiwan	Regional hospital, medical unit, surgical unit, medical-surgical unit, and adult	Nurses	Partial	Pre-post	Questionnaire	-	TAM questionnaire: perceived usefulness, perceived ease of use, attitude to use, and	3 months

				intensive care unit						behavioural intention	
	Jenssen[29]	2016	USA	Children's hospital, 1 inpatient unit	Residents (on rotation during intervention)	Partial	Cross sectional	Questionnaire	-	Advantages and disadvantages of the CDS, suggested improvements and usability (SUS)	3 months
	Keim-Malpass[30,a]	2018	USA	Academic surgical trauma ICU	Point-of-care clinicians (registered nurses, respiratory therapist, nurse practitioner, attending physician)	Partial	Longitudinal	Focus group	-	Qualitative	3 months
	Mahabee-Gittens[31,a]	2018	USA	Academic paediatric medical center, 5 outpatient urgent care centres	Registered nurses	Partial	Longitudinal	Survey	-	Attitudes and barriers to CDS, CDS usability	3 months
	Rosenthal[32]	2019	USA	Paediatric urban teaching hospitals, 2 general EDs	Physicians and Advanced Practice Providers	Partial	Pre-post	Survey	Self-reported use	Knowledge of CDS, impact on clinical decision making, and education requirements	3 months

	Sauro[19,a]	2019	Canada	(as above)				Interviews	-	Qualitative	3 months
	Yoon[33]	2023	South Korea	Tertiary academic hospital, ED	Emergency physicians	Partial	Cross sectional	Survey	-	User experience and system usability survey: effectiveness, efficiency, safety, satisfaction, and reliability	3 months
4-5 months	Feldstein[34]	2023	USA	6 EDs across 2 hospital systems	ED providers	Partial	Cross sectional	Survey	-	User experience	4 months
	Keim-Malpass[30,a]	2018	USA	*(as above)*						Qualitative	4 months
	Mahabee-Gittens[31,a]	2018	USA	*(as above)*				Interviews		Qualitative	4 months
	Suresh[35]	2022	USA	Tertiary care paediatric hospital, ED	Nurses	Partial	Cross sectional	Survey	-	Feedback survey	4 months

5-6 months	Ginestra[36]	2019	USA	Academic hospital, non-ICU inpatient services	Registered nurses, physicians or advanced practitioners	Full	Longitudinal	Survey	-	Perceptions of CDS regarding: the patient's condition; new information discovered at the time of alert; whether and how the alert changed management; and whether and how the alert was useful and/or improved patient care	5-6.5 months
	Holroyd-Leduc[37]	2010	Canada	2 teaching hospitals, orthopaedic wards	Nurses	Partial	Cross sectional	Focus groups	-	Qualitative	5 months
	Rabinovich[38]	2022	Argentina	University hospital, ED	Emergency physicians and radiology residents	Partial	Cross sectional	Survey, interviews	-	System Usability Scale (SUS): Actual use, perceived usefulness, perceived ease of use, output quality; and qualitative	5 months
6-7 months	Bellodi[39]	2017	Italy	3 hospitals: 2 acute care local hospitals in same network (cardiology and medicine wards), 1 teaching hospital	Nurses, physicians	Partial	Pre post	Questionnaire	-	User satisfaction survey	6 months

				(medicine ward)							
	English[40]	2017	USA	3 hospitals, clinical pharmacies	Clinical pharmacists	Full	Cross sectional	Questionnaire	-	UTAUT questionnaire: performance expectancy, effort expectancy, social influence, facilitating conditions, behavioural intentions and usage behaviour	6 months
	Hoekstra[41]	2010	Netherlands	Tertiary university teaching hospital, surgical ICU and thoracic-surgical ICU	Nurses	Partial	Pre-post	Questionnaire	Compliance with recommended pump rates	-	6 months
	Jones[42]	2019	USA	4 urban hospitals, EDs	ED clinicians	Partial	Cross sectional	Survey	-	Usability	6 months
	Uppot[43]	2022	USA	Urban tertiary care referral facility, 2 ICUs (surgical and medical)	ICU staff (not further specified)	Partial	Cross sectional	Survey	-	User acceptance	6 months
7-12 months	Agostini[44]	2008	USA	Academic medical center, hospital wide	Physicians	Full	Cross sectional	Interviews	-	Qualitative	12 months

	Bell[45]	2019	UK	Teaching hospital, 3 cardiac wards	Medical and nurse prescribers	Full	Cross sectional	Interviews, observations	Observations of alert use: frequency and types of alerts, who received alerts and contextual information, for ward rounds and non-ward round prescribing	Qualitative	1 year
	Cho[46]	2013	South Korea	University teaching hospital, two inpatient surgical ICUs	Nurses	Partial	Pre-post	Questionnaire	-	-	7 months
	Groshaus[47]	2012	Canada	2 hospitals, 4 medical units	Nurses	Partial	Stepped wedge	Interviews	-	Qualitative	9 months
	Jauk[48]	2021	Austria	Regional public hospital, 8 participating departments (5 included in study)	Physicians and nurses	Partial	Cross sectional	Questionnaire	Self-reported use	TAM questionnaire: perceived usefulness, perceived ease of use, attitude to use, and behavioural intention	7 months
	Lytle[49]	2015	USA	University hospital, 16 adult units (2 low performing surgical and medical units pre CDS included in the study)	Registered nurses	Partial	Pre-post	Focus groups	-	Qualitative	11 months

	Neame[50]	2021	UK	Regional specialist children's hospital, inpatient wards excluding paediatric ICU	Trainee doctors, consultants, specialist nurse prescribers, prescribing pharmacists	Partial	Pre-post	Questionnaire	-	Acceptability and usability	9 months
	Nydert[51]	2017	Sweden	NR, 3 paediatric wards	Paediatricians	Full	Cross sectional	Interviews	-	Qualitative	1 year
	Pirnejad[52]	2011	Netherlands	Tertiary academic hospital, hematology and oncology inpatient and outpatient departments	Physicians and nurses	Full	Cross sectional	Interviews	-	Qualitative	1 year
	Salwei[53,b]	2021	USA	Academic hospital, ED	Medical residents and attending physicians	Full	Cross sectional	Interviews	-	Qualitative	9-12 months
	Salwei[54,b]	2023	USA	Academic health system, ED	Emergency physicians	Full	Cross sectional	Interviews	-	Qualitative	9 months
	Stutman[55]	2007	USA	6 community hospitals, hospital wide	Providers and pharmacists	Partial	Longitudinal	System data	Alert acceptance (event level)	-	7-8 months
	Henry[56]	2022	USA	Acute-case non teaching hospital, ED and all medical and surgical units	Physicians and nurses (ED, critical care and general ward)	Full	Cross sectional	Interviews	-	Qualitative	7 months

1-2 years	Bersani[57]	2020	USA	Acute care hospital, 12 units	Nurses, patient care assistance, nursing students, attending physicians, PAs, nurse practitioners, fellows, residents, medical students, unit leadership staff, pharmacists, and other	Partial	Cluster randomised stepped wedge trial	Survey	-	Usability: quality of work life, perceived usefulness, perceived ease of use, and user control	18-21 months
	Eden[58]	2020	Australia	Public tertiary care university hospital, hospital wide	Doctors, nurses, pharmacists and allied health professionals	Partial	Cross sectional	Interviews, focus groups	-	Qualitative	14-16 months
	Frymoyer[59]	2020	USA	Academic quaternary-care children's hospital, NICU and PICU	Clinical pharmacists	Partial	Cross sectional	Questionnaire	-	Satisfaction, perceived usability and overall clinical experience	15 months
	Goldstein[60]	2022	USA	Outpatient practices	Opthamologists	Partial	Cross sectional	Survey	-	User experience survey	17 months
	Hum[61]	2014	USA	2 academically affiliated hospitals, both NICUs	Neonatal attending physicians, pediatric residents, neonatology fellows, house physicians, and nurse practitioners	Partial	Cross sectional	Survey	-	User awareness and acceptance, ease of use, recommendations of additional features	12-14 months

	Salwei[62]	2022	USA	Academic health system, ED	Emergency physicians (residents, fellows and attendings)	-	Cross sectional	Survey	-	Computer system usability questionnaire	12-13 months
	Scheepers-Hoeks[63]	2013	Netherlands	Secondary care teaching hospital, ICU	Intensivists, junior doctors and nurse practitioners	Partial	RCT	Survey	-	Satisfaction and suitability of different types of CDS	17 months
	Short[64]	2021	USA	Quaternary-care hospital, ED and medical, surgical, cardiothoracic, cardiac, and neurological ICUs	Resident physicians, nurse practitioners (NP), and physician assistants (PA)	Partial	Pre post	Focus groups	-	Qualitative	13 months
	Zhai[65]	2022	China	Tertiary hospital, 4 medical-surgical wards	Nurses	Full	Cross sectional	Observations and interviews	Observations of use	Qualitative	13 months - 18 months
	Chow[66,a]	2016	Singapore	Tertiary care academic hospital, hospital wide	Physicians	Partial	Longitudinal	System data	Proportion of CDS completed launches for guidance, launches via autotrigger and acceptance of CDS recommendations (event level)	-	20-24 months
2-5 years	Campion[67]	2011	USA	Academic urban tertiary care hospital, surgical and trauma ICUs	Nurses	Partial	Cross sectional	Interviews, observations	Observations of use	Qualitative	4 years 4 months - 5 years 4 months

	Chow[68,b]	2015	Singapore	Adult tertiary hospital, hospital wide	Junior and senior physicians	Full	Cross sectional	Focus groups, questionnaire	-	Qualitative, and survey on situations for use, the perceived credibility and usefulness, and the desired useful features of CDS	3 years 5-7 months
	Chow[66,a,b]	2016	*(as above)*								2 years - 3 years 8 months
	Galanter[69]	2010	USA	Academic urban tertiary hospital, hospital wide	Medical residents, pharmacists (faculty and residents), registered nurses, attending physicians, and other (student, clinical nurse midwife, unknown)	Full	Cross sectional	System data	Alert acceptance (event level)	-	4-5 years
	Lichtner[70]	2020	Australia	Tertiary paediatric hospital, oncology unit	Clinicians in the oncology unit e.g. oncologists, registered nurses, pharmacists	Partial	Cross sectional	Interviews	-	Qualitative	2 years-2 years 9 months

	Lin[71]	2010	Taiwan	University hospital, hospital wide	Physicians	Full	Cross sectional	System data	Percentage of appropriate indications chosen from CDS (appropriate transfusion order is defined as a blood product ordered with an indication, which satisfies the criteria)	-	3 years 4 months - 4 years 3 months
>5 years	Beeler[72]	2016	USA	2 large tertiary care teaching hospitals, outpatient clinics and ambulatory practices	Prescribers e.g. physicians, nurse practitioners, and physician assistants	Full	Cross sectional	System data	Alert acceptance (event level)	-	9-12 years
	Campion[73]	2011	USA	Academic urban tertiary care hospital, surgical and trauma ICUs	Nurses	Full	Cross sectional	Interviews, observations	Observations of use	Qualitative	5 years
	Choi[74]	2019	Korea	Tertiary teaching hospital, hospital wide	Physicians	Partial	Cross sectional	System data	Alert acceptance (event level)	-	10 years 8 months-10 months
	Choudhury[75,b]	2022	USA	Academic hospital, hospital wide	Attending physicians, resident physicians, registered nurses	Full	Cross sectional	Survey	-	Modified UTAUT survey: Expectancy, perceived risk, trust, use of BUC	5+ years

	Choudhury[76,b]	2023	USA	Academic hospital, hospital wide	Attending physicians, resident physicians, registered nurses	Full	Cross sectional	Survey	-	Modified UTAUT survey: Cognitive workload, perceived risk, trust, intention to use, situation awareness	5+ years
	Luna[77]	2017	Argentina	Academic hospital, hospital wide	Physicians with at least 4 years of experience with CPOE system	Partial	Cross sectional	Interviews	-	Qualitative	5+ years (mid 2000's - April 2013/June 2014)
	Ng[78]	2023	Singapore	2 hospitals and outpatient clinics	Physicians, nurses, pharmacists, allied health	Partial	Pre-post	System data	Alert acceptance (event level)	-	5 years 6 months - 6 years 6 months
	Pontefract[79]	2018	UK	Acute hospital, hospital wide	Pharmacists and physicians	Partial	Cross sectional	Focus groups	-	Qualitative	11-12 years
	Van De Sijpe[80]	2022	Belgium	Teaching hospital, hospital wide	Physicians and pharmacists	Full	Cross sectional	Survey, system data	Alert acceptance (event level)	Satisfaction, usefulness, relevance and reasons for overriding severe alerts	10-12 years
	Wong[81]	2017	USA	Urban tertiary care hospital, adult medical, neurology, and surgical ICUs	Providers, pharmacists, nurses	Partial	Pre-post	System data	Alert acceptance (event and patient level)	-	18 years
	Wright[82]	2018	USA	Hospital, outpatient settings	NR	Partial	Pre-post	System data	Alert acceptance (event level)	-	5+ years

ICU Intensive care unit, *ED* emergency department, *CDS* clinical decision support, *TAM* technology acceptance model, *UTAUT* unified theory of acceptance and use of technology, *NICU* neonatal intensive care unit, *PICU* paediatric intensive care unit, *RCT* randomised controlled trial, *NR* not reported, *CPOE* computerised provider order entry system

[a]studies separated for analysis of factors over time

[b]studies combined for analysis of factors over time

Supplementary Note 3. Detailed quality appraisal results

As data were extracted only from study results that met inclusion criteria, the study design was selected and quality was assessed, based only on the methods and results of the study that were eligible for inclusion in the review. For example, a study evaluating both clinical outcomes using patient data and clinicians' perceptions using interviews, would be assessed as a qualitative study rather than mixed methods, given clinical outcomes were not eligible for inclusion in the review. All studies were assessed using the Mixed Methods Appraisal Tool (MMAT).

Supplementary Table 5. Quality appraisal using the MMAT

Timeframe	First author	Year	*Study design (MMAT)*	SQ1	SQ2	Q1	Q2	Q3	Q4	Q5	*Total yes*	*Total no*	*Total CT*
0-1 months	Castellanos[16,a]	2018	Mixed methods	Yes	Yes	Yes	No	Yes	No	No	**2**	**3**	**0**
	Grauer[17]	2022	Quantitative descriptive	Yes	Yes	Yes	CT	Yes	Yes	Yes	**4**	**0**	**1**
1-2 months	Castellanos[16,a]	2018	*(as above)*										
	Guidi[18]	2015	Quantitative descriptive	Yes	Yes	Yes	Yes	Yes	Yes	Yes	**5**	**0**	**0**
	Sauro[19,a]	2019	Mixed methods	Yes	Yes	Yes	Yes	Yes	Yes	Yes	**5**	**0**	**0**
	Tsai[20]	2022	Quantitative descriptive	Yes	Yes	CT	CT	CT	CT	CT	**0**	**0**	**5**
2-3 months	Castellanos[16,a]	2018	*(as above)*										
	DeBie[21]	2021	Mixed methods	Yes	Yes	Yes	Yes	Yes	Yes	Yes	**5**	**0**	**0**
	Harrison[22]	2017	Quantitative descriptive	Yes	Yes	CT	Yes	CT	Yes	Yes	**3**	**0**	**2**
	Petersen[23]	2020	Quantitative descriptive	Yes	Yes	Yes	Yes	CT	Yes	CT	**3**	**0**	**2**
	Thayer[24]	2021	Qualitative	Yes	Yes	Yes	No	No	No	No	**1**	**4**	**0**
3-4 months	Berge[25]	2023	Quantitative descriptive	Yes	Yes	Yes	Yes	Yes	Yes	Yes	**5**	**0**	**0**
	Casey[26]	2023	Mixed methods	Yes	Yes	Yes	Yes	Yes	Yes	Yes	**5**	**0**	**0**
	Chadwick[27]	2017	Qualitative	Yes	Yes	Yes	No	No	No	No	**1**	**4**	**0**
	Huang[28]	2020	Quantitative descriptive	Yes	Yes	Yes	Yes	Yes	Yes	Yes	**5**	**0**	**0**
	Jenssen[29]	2016	Quantitative descriptive	Yes	Yes	CT	Yes	Yes	Yes	Yes	**4**	**0**	**1**
	Keim-Malpass[30,a]	2018	Qualitative	Yes	Yes	Yes	Yes	Yes	Yes	No	**4**	**1**	**0**

	Mahabee-Gittens[31,a]	2018	Mixed methods	Yes	Yes	Yes	Yes	No	No	Yes	**3**	**2**	**0**
	Rosenthal[32]	2019	Quantitative descriptive	Yes	Yes	Yes	CT	CT	No	CT	**1**	**1**	**3**
	Sauro[19,a]	2019	*(as above)*										
	Yoon[33]	2023	Quantitative descriptive	Yes	Yes	Yes	Yes	Yes	Yes	Yes	**5**	**0**	**0**
4-5 months	Feldstein[34]	2023	Quantitative descriptive	Yes	Yes	Yes	Yes	No	Yes	Yes	**4**	**1**	**0**
	Keim-Malpass[30,a]	2018	*(as above)*										
	Mahabee-Gittens[31,a]	2018	*(as above)*										
	Suresh[35]	2022	Quantitative descriptive	Yes	Yes	Yes	Yes	Yes	Yes	Yes	**5**	**0**	**0**
5-6 months	Ginestra[36]	2019	Quantitative descriptive	Yes	Yes	Yes	Yes	Yes	Yes	Yes	**5**	**0**	**0**
	Holroyd-Leduc[37]	2010	Qualitative	Yes	Yes	Yes	Yes	No	No	No	**2**	**3**	**0**
	Rabinovich[38]	2022	Mixed methods	Yes	Yes	CT	Yes	Yes	Yes	No	**3**	**1**	**1**
6-7 months	Bellodi[39]	2017	Quantitative descriptive	Yes	Yes	CT	CT	Yes	CT	CT	**1**	**0**	**4**
	English[40]	2017	Quantitative descriptive	Yes	Yes	Yes	Yes	Yes	Yes	Yes	**5**	**0**	**0**
	Hoekstra[41]	2010	Quantitative descriptive	Yes	Yes	No	No	Yes	CT	Yes	**2**	**2**	**1**
	Jones[42]	2019	Quantitative descriptive	Yes	Yes	CT	CT	Yes	Yes	Yes	**3**	**0**	**2**
	Uppot[43]	2022	Quantitative descriptive	Yes	Yes	Yes	CT	Yes	CT	No	**2**	**1**	**2**
7-12 months	Agostini[44]	2008	Qualitative	Yes	Yes	Yes	Yes	Yes	Yes	Yes	**5**	**0**	**0**
	Bell[45]	2019	Mixed methods	Yes	Yes	Yes	Yes	Yes	Yes	Yes	**5**	**0**	**0**
	Cho[46]	2013	Quantitative descriptive	Yes	Yes	Yes	Yes	Yes	Yes	Yes	**5**	**0**	**0**
	Groshaus[47]	2012	Qualitative	Yes	Yes	Yes	Yes	No	No	No	**2**	**3**	**0**
	Jauk[48]	2022	Qualitative	Yes	Yes	Yes	Yes	Yes	Yes	Yes	**5**	**0**	**0**
	Lytle[49]	2021	Quantitative descriptive	Yes	Yes	Yes	Yes	Yes	Yes	Yes	**5**	**0**	**0**
	Neame[50]	2015	Mixed methods	Yes	Yes	Yes	No	No	Yes	CT	**2**	**2**	**1**
	Nydert[51]	2021	Quantitative descriptive	Yes	Yes	CT	CT	CT	CT	Yes	**1**	**0**	**4**
	Pirnejad[52]	2017	Qualitative	Yes	Yes	Yes	Yes	Yes	Yes	Yes	**5**	**0**	**0**
	Salwei[53,b]	2011	Qualitative	Yes	Yes	Yes	Yes	Yes	No	Yes	**4**	**1**	**0**

	Salwei[54,b]	2021	Qualitative	Yes	Yes	Yes	Yes	Yes	Yes	Yes	**5**	**0**	**0**
	Stutman[55]	2023	Qualitative	Yes	Yes	Yes	Yes	Yes	Yes	Yes	**5**	**0**	**0**
	Henry[56]	2007	Quantitative descriptive	Yes	Yes	CT	Yes	Yes	Yes	No	**3**	**1**	**1**
1-2 years	Bersani[57]	2020	Mixed methods	Yes	Yes	Yes	Yes	Yes	Yes	Yes	**5**	**0**	**0**
	Eden[58]	2020	Qualitative	Yes	Yes	Yes	Yes	Yes	Yes	Yes	**5**	**0**	**0**
	Frymoyer[59]	2020	Quantitative descriptive	Yes	Yes	Yes	Yes	Yes	Yes	No	**4**	**1**	**0**
	Goldstein[60]	2022	Quantitative descriptive	Yes	Yes	Yes	CT	No	CT	Yes	**2**	**1**	**2**
	Hum[61]	2014	Quantitative descriptive	Yes	Yes	Yes	Yes	CT	No	CT	**2**	**1**	**2**
	Salwei[62]	2022	Quantitative descriptive	Yes	Yes	Yes	Yes	Yes	Yes	Yes	**5**	**0**	**0**
	Scheepers-Hoeks[63]	2013	Quantitative descriptive	Yes	Yes	Yes	Yes	CT	Yes	CT	**3**	**0**	**2**
	Short[64]	2021	Qualitative	Yes	Yes	Yes	Yes	No	Yes	No	**3**	**2**	**0**
	Zhai[65]	2022	Qualitative	Yes	Yes	Yes	Yes	Yes	Yes	Yes	**5**	**0**	**0**
	Chow[66,a]	2016	Quantitative non randomised	Yes	Yes	Yes	Yes	No	Yes	Yes	**4**	**1**	**0**
2-5 years	Campion[67]	2011	Qualitative	Yes	Yes	Yes	Yes	No	No	No	**2**	**3**	**0**
	Chow[68,b]	2015	Mixed methods	Yes	Yes	Yes	Yes	Yes	Yes	Yes	**5**	**0**	**0**
	Chow[66,a,b]	2016	*(as above)*										
	Galanter[69]	2010	Quantitative descriptive	Yes	Yes	Yes	Yes	Yes	Yes	Yes	**5**	**0**	**0**
	Lichtner[70]	2020	Qualitative	Yes	Yes	Yes	Yes	Yes	Yes	Yes	**5**	**0**	**0**
	Lin[71]	2010	Quantitative descriptive	Yes	Yes	Yes	Yes	Yes	Yes	CT	**4**	**0**	**1**
>5 years	Beeler[72]	2016	Quantitative descriptive	Yes	Yes	Yes	Yes	Yes	Yes	Yes	**5**	**0**	**0**
	Campion[73]	2011	Qualitative	Yes	Yes	Yes	Yes	Yes	Yes	Yes	**5**	**0**	**0**
	Choi[74]	2019	Quantitative descriptive	Yes	Yes	Yes	Yes	Yes	Yes	Yes	**5**	**0**	**0**
	Choudhury[75,b]	2023	Quantitative descriptive	Yes	Yes	Yes	Yes	Yes	Yes	Yes	**5**	**0**	**0**
	Choudhury[76,b]	2022	Quantitative descriptive	Yes	Yes	Yes	Yes	Yes	Yes	Yes	**5**	**0**	**0**
	Luna[77]	2017	Qualitative	Yes	Yes	Yes	CT	CT	Yes	No	**2**	**1**	**2**

	Ng[78]	2023	Quantitative descriptive	Yes	Yes	Yes	Yes	Yes	Yes	Yes	**5**	**0**	**0**
	Pontefract[79]	2018	Qualitative	Yes	Yes	Yes	Yes	Yes	Yes	Yes	**5**	**0**	**0**
	Van De Sijpe[80]	2022	Quantitative descriptive	Yes	Yes	Yes	CT	Yes	No	Yes	**3**	**1**	**1**
	Wong[81]	2017	Quantitative descriptive	Yes	Yes	Yes	Yes	No	Yes	Yes	**4**	**1**	**0**
	Wright[82]	2018	Quantitative non randomised	Yes	Yes	CT	Yes	Yes	No	Yes	**3**	**1**	**1**

MMAT Mixed Methods appraisal tool, *SQ* MMAT screening question, *Q* MMAT question, *CT* can't tell

[a]studies separated for analysis of factors over time

[b]studies combined for analysis of factors over time

Supplementary Figure 1. Barriers and facilitators identified in CFIR domains 1-6 months following CDS implementation

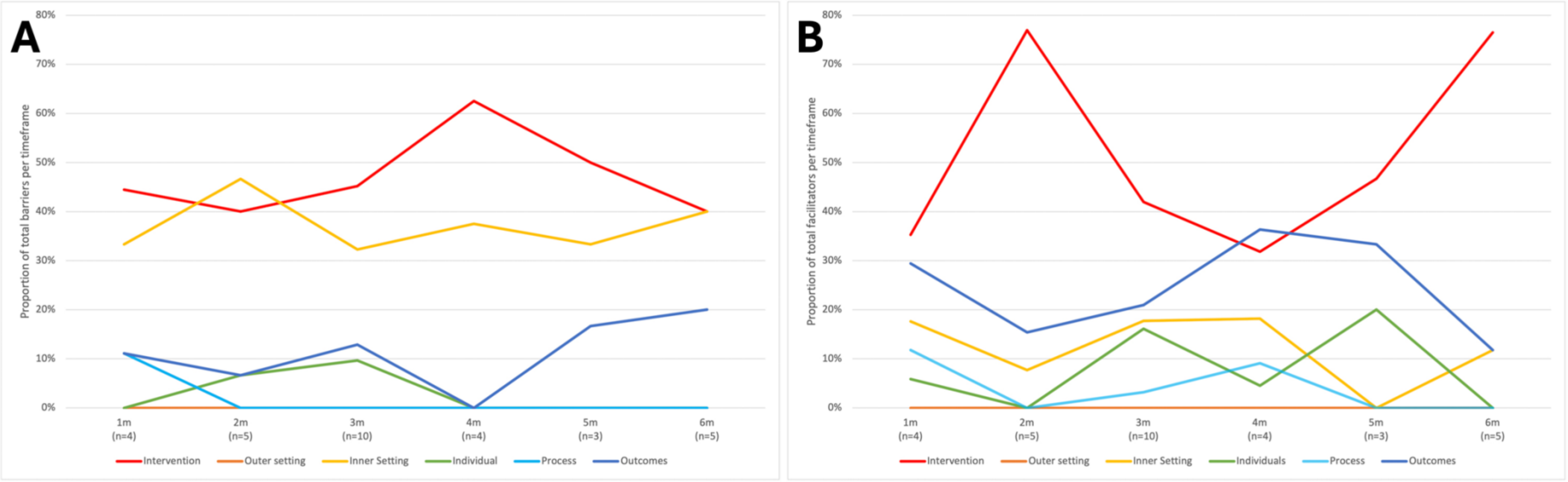

Figure legend: *m* months, *n* number of study time-points.

A) The proportion of **barriers** identified in each CFIR domain are presented relative to the total number of barriers identified at monthly intervals in the first 6 months following CDS implementation. Barriers were counted once per study time-point (see factor count calculation in Supplementary Table 3) and summed across all study time-points within each monthly interval. Studies conducted between 0-1 month (n=2) were excluded from this figure as no barriers were reported during this time.

B) The proportion of **facilitators** identified in each CFIR domain are presented relative to the total number of facilitators identified at monthly intervals in the first 6 months following CDS implementation. Facilitators were counted once per study time-point (see factor count calculation in Supplementary Table 3) and summed across all study time-points within each monthly interval. Studies conducted between 0-1 month (n=2) were excluded from this figure as only one facilitator was reported during this time.

Supplementary Table 6. Barriers and facilitators identified in key constructs 1-6 months following CDS implementation

CFIR Domain	CFIR Construct	Timeframe					
		1 month	2 months	3 months	4 months	5 months	6 months
Intervention	Complexity			• Easy to use (+)<br>• **Easy to learn (+)**	• Easy to use (+)<br>• Time and effort to complete (+)	• Ease of use (+/-)	• Easy to use (+)<br>• Time and effort (-/+)
	Data Quality			• Recommendation quality (+)			
	Design Quality and Packaging		• Integration with other CIS (-)	• **Rule or algorithm design (-)**<br>• **Interface design (-/+)**<br>• **Visibility of patient status (-)**	• Integration with other CIS (-)		
	Relative Advantage	• Usefulness (+/-)	• **Efficiency (+/-)**<br>• **Valued system features (+)**	• Usefulness (+/-)<br>• **System performance (+)**<br>• **Satisfied (+)**		• Usefulness/utility (-/+)	• Usefulness (+/-)<br>• **Preferred (+/-)**
	Evidence strength and quality						
Inner Setting	Available Resources			• **Resources to support system use (-/+)**			
	Compatibility			• Workflow fit (+/-)	• Workflow fit (+/-)	• Workflow fit (-)	
	Task and Work Context						

Individuals	Individual stage of change						
	Self-Efficacy			• **Poor understanding and skills (-)**			
	Knowledge and beliefs about the intervention			• **Attitude to using (+)**<br>• **Intention to use (+)**			
	Individual stage of change			• **Ongoing use (+)**			
Outcomes	Innovation Deliverers			• **Improved confidence (+)**<br>• **Improved clinical decision making (+)**<br>• **Improved/reduced efficiency (+/-)**	• **Prompts consideration (+)**		
	Innovation Receivers	• **Timeliness (+)**<br>• Improved patient care (+)		• Improved patient care (+)<br>• **Improved patient communication (+)**<br>• **Improved or reduced safety (+/-)**		• Improved patient care (+)	

CFIR Consolidated Framework for Implementation Research, *CIS* Clinical Information System.

Note: Key constructs presented in this table were identified as barriers or facilitators in over 25% of study time-points within a given timeframe. Factors reported were identified in 2 or more studies within each timeframe where (+) indicates a facilitator to acceptance and use i.e. positive direction, and (-) indicates a barrier to acceptance and use i.e. negative direction. Factors in **bold** were uniquely reported within a particular timeframe.

Supplementary Table 7. Completed PRISMA 2020 Checklist

Section and Topic	Item #	Checklist item	Location where item is reported
**TITLE**			
Title	1	Identify the report as a systematic review.	P1
**ABSTRACT**			
Abstract	2	See the PRISMA 2020 for Abstracts checklist.	P1
**INTRODUCTION**			
Rationale	3	Describe the rationale for the review in the context of existing knowledge.	P1-2
Objectives	4	Provide an explicit statement of the objective(s) or question(s) the review addresses.	P2
**METHODS**			
Eligibility criteria	5	Specify the inclusion and exclusion criteria for the review and how studies were grouped for the syntheses.	P9-10
Information sources	6	Specify all databases, registers, websites, organisations, reference lists and other sources searched or consulted to identify studies. Specify the date when each source was last searched or consulted.	P9
Search strategy	7	Present the full search strategies for all databases, registers and websites, including any filters and limits used.	P9 & Supplementary Materials
Selection process	8	Specify the methods used to decide whether a study met the inclusion criteria of the review, including how many reviewers screened each record and each report retrieved, whether they worked independently, and if applicable, details of automation tools used in the process.	P10
Data collection process	9	Specify the methods used to collect data from reports, including how many reviewers collected data from each report, whether they worked independently, any processes for obtaining or confirming data from study investigators, and if applicable, details of automation tools used in the process.	P10
Data items	10a	List and define all outcomes for which data were sought. Specify whether all results that were compatible with each outcome domain in each study were sought (e.g. for all measures, time points, analyses), and if not, the methods used to decide which results to collect.	P10
	10b	List and define all other variables for which data were sought (e.g. participant and intervention characteristics, funding sources). Describe any assumptions made about any missing or unclear information.	P10
Study risk of bias assessment	11	Specify the methods used to assess risk of bias in the included studies, including details of the tool(s) used, how many reviewers assessed each study and whether they worked independently, and if applicable, details of automation tools used in the process.	P10
Effect measures	12	Specify for each outcome the effect measure(s) (e.g. risk ratio, mean difference) used in the synthesis or presentation of results.	P10-11

Section and Topic	Item #	Checklist item	Location where item is reported
Synthesis methods	13a	Describe the processes used to decide which studies were eligible for each synthesis (e.g. tabulating the study intervention characteristics and comparing against the planned groups for each synthesis (item #5)).	P10
	13b	Describe any methods required to prepare the data for presentation or synthesis, such as handling of missing summary statistics, or data conversions.	P10-11
	13c	Describe any methods used to tabulate or visually display results of individual studies and syntheses.	P11
	13d	Describe any methods used to synthesize results and provide a rationale for the choice(s). If meta-analysis was performed, describe the model(s), method(s) to identify the presence and extent of statistical heterogeneity, and software package(s) used.	P10-11
	13e	Describe any methods used to explore possible causes of heterogeneity among study results (e.g. subgroup analysis, meta-regression).	N/A
	13f	Describe any sensitivity analyses conducted to assess robustness of the synthesized results.	N/A
Reporting bias assessment	14	Describe any methods used to assess risk of bias due to missing results in a synthesis (arising from reporting biases).	N/A
Certainty assessment	15	Describe any methods used to assess certainty (or confidence) in the body of evidence for an outcome.	N/A
**RESULTS**			
Study selection	16a	Describe the results of the search and selection process, from the number of records identified in the search to the number of studies included in the review, ideally using a flow diagram.	P2
	16b	Cite studies that might appear to meet the inclusion criteria, but which were excluded, and explain why they were excluded.	P8
Study characteristics	17	Cite each included study and present its characteristics.	P2 & Supplementary Materials
Risk of bias in studies	18	Present assessments of risk of bias for each included study.	P3 & Supplementary Materials
Results of individual studies	19	For all outcomes, present, for each study: (a) summary statistics for each group (where appropriate) and (b) an effect estimate and its precision (e.g. confidence/credible interval), ideally using structured tables or plots.	P3-5
Results of syntheses	20a	For each synthesis, briefly summarise the characteristics and risk of bias among contributing studies.	Supplementary Materials
	20b	Present results of all statistical syntheses conducted. If meta-analysis was done, present for each the summary estimate and its precision (e.g. confidence/credible interval) and measures of statistical heterogeneity. If comparing groups, describe the direction of the effect.	P3-5
	20c	Present results of all investigations of possible causes of heterogeneity among study results.	N/A

Section and Topic	Item #	Checklist item	Location where item is reported
	20d	Present results of all sensitivity analyses conducted to assess the robustness of the synthesized results.	N/A
Reporting biases	21	Present assessments of risk of bias due to missing results (arising from reporting biases) for each synthesis assessed.	N/A
Certainty of evidence	22	Present assessments of certainty (or confidence) in the body of evidence for each outcome assessed.	N/A
**DISCUSSION**			
Discussion	23a	Provide a general interpretation of the results in the context of other evidence.	P6-9
	23b	Discuss any limitations of the evidence included in the review.	P8-9
	23c	Discuss any limitations of the review processes used.	P8-9
	23d	Discuss implications of the results for practice, policy, and future research.	P6-9
**OTHER INFORMATION**			
Registration and protocol	24a	Provide registration information for the review, including register name and registration number, or state that the review was not registered.	P9
	24b	Indicate where the review protocol can be accessed, or state that a protocol was not prepared.	P9
	24c	Describe and explain any amendments to information provided at registration or in the protocol.	N/A
Support	25	Describe sources of financial or non-financial support for the review, and the role of the funders or sponsors in the review.	P11
Competing interests	26	Declare any competing interests of review authors.	P11
Availability of data, code and other materials	27	Report which of the following are publicly available and where they can be found: template data collection forms; data extracted from included studies; data used for all analyses; analytic code; any other materials used in the review.	P11

From: Page MJ, McKenzie JE, Bossuyt PM, Boutron I, Hoffmann TC, Mulrow CD, et al. The PRISMA 2020 statement: an updated guideline for reporting systematic reviews. BMJ 2021;372:n71. doi: 10.1136/bmj.n71
